# The epidemiology of unintentional falls among older people in the Middle East and North Africa: a systematic review and meta-analysis

**DOI:** 10.7189/jogh.15.04072

**Published:** 2025-03-14

**Authors:** Karima Chaabna, Anupama Jithesh, Salina Khawaja, Jasmine Aboughanem, Ravinder Mamtani, Sohaila Cheema

**Affiliations:** Weill Cornell Medicine-Qatar, Institute for Population Health, Doha, Qatar

## Abstract

**Background:**

Falls epidemiology in the Middle East and North Africa (MENA) remains underexplored despite being a major cause of morbidity and mortality. We synthesised the data on unintentional falls among older adults in MENA countries.

**Methods:**

We conducted a systematic review, meta-analysis, and meta-regression, searching MEDLINE/PubMed, Web of Science, and Google Scholar up to 5 May 2024, without language or time restriction. We included records on fall prevalence, frequency, location, self-reported reasons, consequences, and health care utilisation. Two reviewers independently conducted multi-stage screening, data extraction, and quality assessment. We estimated the pooled-average prevalence using random-effect models and calculated MENA population-size weighted-averages.

**Results:**

We identified 7392 records, finding 90 eligible studies covering 99 588 older adults from 14 countries. The MENA population-size weighted-average prevalence of older adults with ≥1 fall was 17.6% (95% confidence interval (CI) = 9.8–36.3), with higher prevalence in older age (*P* = 0.001). Among fallers, 59.0% (95% CI = 40.0–76.0) reported ≥2 falls. The pooled prevalence of fallers was 60.1% (95% CI = 42.2–75.7) among older trauma unit patients, while 49.3% (95% CI = 33.9–64.8) of older outpatients reported falling in the past year. Falls occurred primarily at home (pooled-average proportion of fallers = 66.1%; 95% CI = 46.6–81.3), with fewer at work (10.1%; 95% CI = 1.6–44.2), and in hospitals (6.0%; 95% CI = 2.5–13.8). On average, post-fall, 45.6% (95% CI = 37.8–53.5) sought medical care, 36.8% (95% CI = 21.9–54.8) had fractures, and 17.3% (95% CI = 8.0–33.2) experienced anxiety or depression. The in-hospital death rate following a fall was 7.5% (95% CI = 1.5–29.8). Self-reported reasons for falls included medical conditions, balance problems, and environmental factors. We observed substantial heterogeneity and some publication bias (LFK index = 7.34).

**Conclusions:**

The prevalence of older adults in MENA reporting ≥1 fall is lower than global estimates. However, substantial fracture proportions, mental health issues, and in-hospital deaths following a fall underscore the need for region-specific fall prevention strategies.

**Registration:**

This review is registered on the Open Science Framework (https://osf.io/3cu4q; https://doi.org/10.17605/OSF.IO/3CU4Q).

Falls are a substantial global health concern, with 172 million incidents reported annually that lead to disabilities [1]. Falls place a significant strain on health care systems, patients, families, and communities affected [2]. Remarkably, 82% of fall-related deaths occur in low- and middle-income countries [2]. Without targeted interventions, researchers anticipate a drastic increase in fall-related injuries in the coming decades [2,3]. Despite the urgency of addressing this issue, the scarcity of data in these countries complicates understanding geographical disparities [2].

Falls are increasing worldwide with a generally aging global population [2], with older adults facing the highest risk of severe injury or death from unintentional falls [4]. This risk escalates with age [4] due to increasing prevalence of multimorbidity, polypharmacy, and frailty in the older adults [5], leading to hospitalisation, significant functional decline, increased dependence on care, and premature admission to institutional care [6]. Demographic projections indicate that the global older population will double between 2015–2050 [7], with the trend in aging populations becoming more pronounced in low- and middle-income countries [7]. By then, 80% of the world’s older population will reside in these countries [7].

Aligning with global trends, the Middle East and North Africa (MENA) region, which encompasses countries with diverse socioeconomic backgrounds, is experiencing population aging. The older population is expected to grow most rapidly in MENA over the next three decades [8]. Despite this, there is a lack of systematic review analysing fall prevalence, location, self-reported reasons, consequences, and health care utilisation in this region. Only two systematic reviews, with searches up to 2020, have addressed fall prevalence in the MENA region [9,10]. This data gap limits the ability to effectively understand and address fall-related health outcomes in MENA. Our systematic review aims to fill this gap by providing a comprehensive description of the epidemiology of falls among older adults in MENA. We synthesised the available evidence on the prevalence of fallers, frequency of falls, location, self-reported perceived reasons for falling, negative health consequences, and health care utilisation following falls among older individuals.

## METHODS

### Search strategy and selection criteria

Our systematic review, meta-analysis, and meta-regression was developed based on the Cochrane Collaboration Handbook [11] and adheres to the Preferred Reporting Items for Systematic Reviews and Meta-analysis 2020 (PRISMA 2020) [12] guidelines (Table S1 in the **Online Supplementary Document**). The review protocol was preregistered on Open Science Framework (https://osf.io/3cu4q).

We developed the eligibility criteria using the Population, Intervention, Comparison, Outcome, Timing and Setting (PICOTS) framework [13]. The population of interest includes the 57 million [14,15] older adults (aged ≥60, as defined by the United Nations [16]), across the 20 MENA countries. Throughout this manuscript, we use the term ‘older adults’ to refer to these individuals aged ≥60 years. The list of MENA countries is based on definitions by the World Health Organization (WHO), Joint United Nations Programme on HIV/AIDS (UNAIDS), the World bank, and Global Burden of Disease initiative, used in published systematic reviews on population health in MENA [17-25]. This region comprises low-income countries (LICs) (Sudan, Syria, and Yemen), lower-middle-income countries (Algeria, Djibouti, Egypt, Lebanon, Morocco, Pakistan, Palestine, and Tunisia), upper-middle-income countries (Iraq, Jordan, and Libya), and high-income countries (HICs) (Bahrain, Kuwait, Oman, Qatar, Saudi Arabia, and United Arab Emirates) [26]. We included records that include primary data about falls among older men and women in community, clinical, residential care and occupational settings.

We examined falls as defined by the WHO: ‘an event, which results in a person coming to rest inadvertently on the ground or floor or other lower level’ [4]. This definition excludes falls caused by assault, self-harm, animals, burning buildings, transport vehicles, fire, water, and machinery [4]. The primary outcomes of interest were prevalence of older adults reporting ≥1 fall, fall frequency, location, self-reported perceived reasons for falling, negative health consequences of falls, and health care utilisation following a fall. In our study, we did not restrict the time of data collection. We included any study design but excluded case reports and case series.

We conducted extensive searches on PubMed/MEDLINE and Web of Science. We also searched Google Scholar to identify grey literature published in local journals. Our search strategy had no time and language restrictions. We set alerts for ongoing updates and last checked on May 2024. We screened the reference lists of included studies and relevant systematic reviews for potential additional inclusion. We employed free-text terms and controlled vocabulary pertaining to the following concepts: fall, older adults and MENA countries (Box S1–2 in the **Online Supplementary Document**). Our strategy excluded keywords related to outcome measurements (*e.g*. prevalence) to maintain a wide-ranging search approach [11]. An experienced librarian refined our search strategy, optimising the selection of databases and search terms.

Three reviewers (AJ, SK, JA) participated in the screening process. Each paper was screened independently by two of the three reviewers at both the title/abstract and full-text stages. using Rayyan software (Rayyan Systems Inc, Cambridge, MA, USA, https://www.rayyan.ai/) [27]. An additional reviewer (KC) checked the excluded studies. Conflicts were resolved by consensus.

### Data analysis

Two of the three reviewers (AJ, SK, JA) independently extracted data for each study, including:

1. study characteristics (*e.g*. study design, sampling method, data collection time, sample size)

2. setting

3. sociodemographic information (*e.g*. population type, age, sex, and residence)

4. details of the diagnostic tool

5. outcome.

We applied the WHO system approach [2] to classify the outcome of self-reported reasons for falls into person’s biology, physical environment, behaviour, and cultural environment and socioeconomic environment. An additional reviewer (KC) verified the accuracy of all extracted data. Conflicts were resolved by consensus.

Two of the four reviewers (AJ, SK, JA, KC) independently assessed the quality of the studies using the conservative risk of bias in prevalence studies tool [28]. Conflicts were resolved by consensus. Each included study was evaluated for every assessment item and assigned a low or high risk of bias. For the item assessing the primary study’s instrument validity, a high risk of bias was assigned if the study did not use a clinical diagnosis to measure the reported outcomes of interest. If the information was insufficient to judge an item, a high risk of bias was assigned. This conservative approach helps in assessing reporting biases in the included studies. Additionally, conflict of interest was investigated as recommended by the Cochrane Collaboration Handbook [11]. In accordance with COSMOS-E guidance [29], we did not compute a summary quality score.

We adhered to the Grading of Recommendations, Assessment, Development, and Evaluation approach to appraise the certainty in the body of evidence synthesised in the systematic review. We conducted the appraisal through discussions on the validity and reliability of our estimates considering the study-level quality assessment and reporting biases in the body of evidence, the precision of the estimates, the consistency of the primary study results, and how directly the body of evidence answers the research question [30].

We conducted a meta-analysis to estimate the pooled weighted average prevalence and proportion, using random-effect model to account for expected heterogeneity across studies. Details of the study selection for the meta-analysis is provided in the appendix (Box S3 in the **Online Supplementary Document**). Weighted average proportions (*e.g*. for frequency of fall, 1 and ≥2 falls) do not add up to 100% as each weighted average proportion derives from a different set of studies (depending on the available data). The meta-analysis was conducted using the metaprop function from *R*, version 4.0.3 meta package (R Core Team, Vienna, Austria).

The between-study heterogeneity was assessed using Cochrane’s Q statistic to confirm between-study heterogeneity; and *I*^2^ to quantify the magnitude of the variability between-studies that is due to true differences in prevalence/proportion rather than chance. Between-study heterogeneity was considered substantial when *I*^2^>50% [31]. We conducted subgroup meta-analyses and random-effects meta-regressions to investigate sources of heterogeneity related to study methodology (diagnosis method (clinical *vs*. self- reported), age definition, sample size, response rate, time frame, sampling method, year of data collection), social determinants (*e.g*. age, sex, country, and World Bank country-income level), and population types (general population, older adults residing in nursing homes, outpatients, and trauma patients). Subgroup meta-analysis by population type serves as a proxy for analysis by setting type (*e.g*. community, nursing home, and outpatient settings). To address potential recall bias, we stratified our analyses by the timeframe of fall occurrence. We estimated the pooled prevalence of fallers for three distinct categories:

1. current falls identified through medical records of trauma patients in hospitals, which are not subject to recall bias (Figure S5 in the **Online Supplementary Document**)

2. falls reported within the past year among the general population (Table S4 and Figure S2 in the **Online Supplementary Document**), older adults in residential homes (Table S7 and Figure S4 in the **Online Supplementary Document**), and outpatients (Figure S6 in the **Online Supplementary Document**), which may have moderate recall bias

3. falls reported without a specific timeframe (*i.e*. history of falls) among the general older population (Table S5 and Figure S3 in the **Online Supplementary Document**), which may have greater recall bias.

Univariable and multivariable meta-regressions were computed using the metareg function from the *R* meta package. Logit transformation was applied to compute odds ratios (ORs) to quantify the association of between-studies heterogeneity sources with effect size.

We utilised the doi plot, which shows the normal-quantile (z-score on the y-axis) against the logit transformation of prevalence (x-axis), and the LFK index to assess publication bias by visualising asymmetry and quantifying potential asymmetry of study effects [32]. An LFK index less than −1 or greater than +1 indicates publication bias. If publication bias was detected in the meta-analysis, we addressed this by computing the 95% prediction interval of the corresponding pooled prevalence. This approach provided a range of prevalence values likely to contain the true value, accounting for potential biases and uncertainties in the estimation process [33,34]. We did not use the typical funnel plot, Egger's regression or trim-and-fill method to assess publication bias due to methodological issues in their application to proportion studies [35].

We estimated missing data collection years by adjusting the publication year using the median difference between publication and data collection, calculated from studies with complete data.

To calculate the MENA regional prevalence of older adults reporting ≥1 falls, we used a population-size weighted average approach. We first estimated the number of older adults reporting ≥1 fall in the past year for each country, using pooled prevalence data from our meta-analyses and the UN 2022 population size data for the older (≥60-year old) population [14,15]. For countries without prevalence data measured within the past year, we used a hierarchical approach:

1. country-specific pooled prevalence combining past one year and history of falls data, when available

2. if unavailable, country-specific pooled prevalence of history of falls, when available

3. if unavailable, the pooled prevalence from the corresponding World Bank income level measured within the past year.

For Palestine, due to data limitations, we used six-month fall data. This approach was based on the assumption that the difference between the prevalence of older adults reporting ≥1 falls in the past one year and the prevalence of older adults with a history of ≥1 falls was similar. This assumption was supported by our meta-regression analysis, which found no statistical difference between past-year fall prevalence and history of falls (*P* > 0.05). We also assumed that countries within the same World Bank income level would have similar fall prevalence due to shared socioeconomic and cultural characteristics, and their fall prevalence would approximate the average prevalence calculated for that income level. The type of pooled prevalence used is detailed in **Table 1**. This approach ensures that the regional prevalence reflects the entire region by proportionally accounting for each population, resulting in a more representative regional figure.

**Table 1:** Population-size weighted prevalence of fallers among the older adults in the MENA region

		Random-effect meta-analysis*				Estimated number of older adults reporting ≥1 fall			Population-size weighted prevalence		
	**Older population size [** **14** **,** **15** **]**	**Pooled prevalence (%)**	**95% CI**		**Geographical coverage and time frame**	**Number of fallers**	**95% CI**		**%**	**95% CI**	
			
			**Lower**	**Upper**			**Lower**	**Upper**		**Lower**	**Upper**
**Low-income countries**	5 565 115	-	-	-	-	89 042	66 781	111 302	1.6	1.2	2.0
Sudan†	2 574 992	1.6	1.2	2.0	Country, 1y‡	41 200	30 900	51 500	-	-	-
Syrian Arab Republic	1 603 193	1.6	1.2	2.0	WBC, 1y‡	25 651	19 238	32 064	-	-	-
Yemen	1 386 930	1.6	1.2	2.0	WBC, 1y‡	22 191	16 643	27 739	-	-	-
**Lower-middle-income countries**	36 150 185	-	-	-	-	8 583 818	4 727 578	18 391 115	23.7	13.1	50.9
Algeria	4 342 130	29.4	14.8	50.1	WBC, HF/1y¶	1 276 586	642 635	2 175 407	-	-	-
Djibouti	79 649	29.4	14.8	50.1	WBC, HF/1y¶	23 417	11 788	39 904	-	-	-
Egypt	8 562 126	40.8	32.3	49.8	Country, 1y‡	3 493 347	2 765 567	4 263 939	-	-	-
Lebanon	790 999	29.4	14.8	50.1	WBC, HF/1y¶	232 554	117 068	396 290	-	-	-
Morocco	4 446 216	29.4	14.8	50.1	WBC, HF/1y¶	1 307 188	658 040	2 227 554	-	-	-
State of Palestine	290 472	38.1	31.2	45.5	Country, 6m§	110 670	90 627	132 165	-	-	-
Pakistan†	15 946 019	10.3	1.2	52.1	Country, HF	1 642 440	191 352	8 307 876	-	-	-
Tunisia	1 692 574	29.4	14.8	50.1	WBC, HF/1y¶	497 617	250 501	847 980	-	-	-
**Upper-middle-income countries**	3 509 571	-	-	-	-	508 382	318 758	784 651	14.5	9.1	22.4
Iraq	2 272 003	6.6	3.7	10.6	Country, HF	149 952	84 064	240 832	-	-	-
Jordan	711 378	35.0	27.0	43.9	Country, 1y‡	248 982	192 072	312 295	-	-	-
Libya	526 190	20.8	8.1	44.0	WBC, HF/1y¶	109 448	42 621	231 524	-	-	-
**High-income countries**	3 064 484	-	-	-	-	825 394	463 582	1 344 053	26.9	15.1	43.9
Bahrain	101 086	28.5	18.2	41.6	WBC, 1y‡	28 810	18 398	42 052	-	-	-
Kuwait	394 948	28.5	18.2	41.6	WBC, 1y‡	112 560	71 881	164 298	-	-	-
Oman	204 058	28.5	18.2	41.6	WBC, 1y‡	58 157	37 139	84 888	-	-	-
Qatar	89 466	28.5	18.2	41.6	WBC, 1y‡	25 498	16 283	37 218	-	-	-
Saudi Arabia†	1 947 494	23.8	11.6	42.7	Country, 1y‡	463 504	225 909	831 580	-	-	-
United Arab Emirates	327 432	41.8	28.7	56.2	Country, 1y‡	136 867	93 973	184 017	-	-	-
Middle East and North Africa	56 851 481	-	-	-	MENA region	10 006 636	5 576 699	20 631 121	17.6	9.8	36.3

CI – confidence interval, HF – history of fall, WB – the World Bank income level

*Meta-analysis forest plots showing pooled prevalence of fallers among the older population living in community by country are presented in Figure S2–3 in the **Online Supplementary Document**.

†Including data from national surveys.

‡1y – past one year.

§6m – past six months.

¶HF/1y indicates that pooled prevalence was computed utilising datapoints with both time frames.

## RESULTS

**Figure 1:**
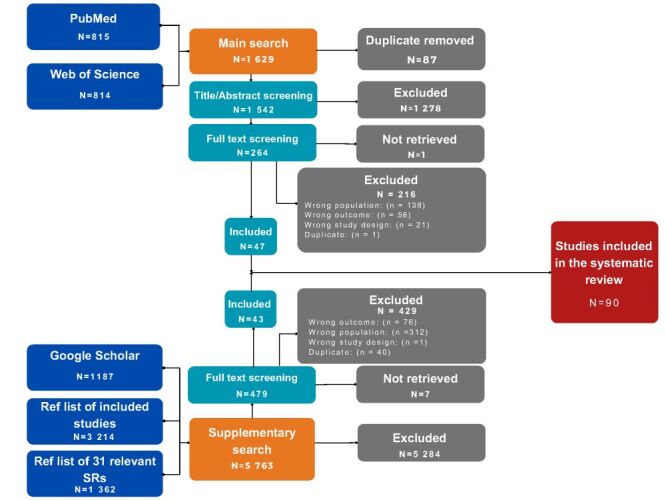
PRISMA flow diagram of the study selection. PRISMA – Preferred Reporting Items for Systematic Reviews and Meta-analysis.

We included 90 articles from the initial 7392 identified, featuring data on 99 588 older adults from 14 countries. The primary study selection flow diagram is presented in **Figure 1** while the characteristics of the included studies are presented in Table S2 in the **Online Supplementary Document**. Among the included studies, 79 reported the prevalence of older adults reporting ≥1 fall, 24 reported fall frequency, 15 investigated the self-reported perceived reasons of falling, and 40 examined the consequences of falls among the older population. The included studies contributed to 740 extracted outcome measures stratified by sex, age group, setting, population type, time frame, and frequency. Additionally, we identified, nationwide survey in community-dwelling population for Pakistan, Saudi Arabia, and Sudan, as well as registry-based studies from Kuwait, Qatar, and the UAE.

A summary and detailed study-level quality assessment are provided in the appendix (Figure S1 and Table S3 in the **Online Supplementary Document**). Overall, studies demonstrated good internal validity, with consistent data collection methods, appropriate time frames for measuring falls, and correct calculations of prevalence. Additionally, over 98% of the studies collected data directly from participants or medical records. Although all included studies aimed to assess unintentional falls, only 42.2% provided a clear definition of falls and 44.4% used a simple question about history of falls, which may introduce recall bias. The external validity was difficult to assess as 60.0% of the studies did not report response rates. Additionally, in 99.0% of the studies, the population sample was closely representative of their target populations, yet only 50.0% had national coverage.

We estimated the MENA population-size-weighted average prevalence of older adults living in community setting reporting ≥1 fall at 17.6%, totalling 10.0 million individuals (**Table 1**). The prevalence varies by income level, with 26.9% in HICs and 1.6% in LICs. The prevalence in LICs was derived from the Sudan National Household Health Survey, which utilised multistage cluster random sampling [36]. At the country level, the numbers of older adults experiencing falls ranged from 23 417 in Djibouti, to 3.5 million in Egypt.

We identified a significant increase in the pooled prevalence of falls in the past year among adults ≥70 years old compared to those aged 60–69 years (Table S4–5 in the **Online Supplementary Document**). We found significant differences between World Bank income groups and countries (Figure S2–3 in the **Online Supplementary Document**). However, our subgroup meta-analyses revealed no statistically significant sex-specific differences in falls prevalence and no time trend.

We also found no statistically significant difference between the proportions of older fallers reporting one fall (weighted average proportions (WAP) = 54.0%; 95% confidence interval (CI) = 46.0–61.0%, n (number of studies) = 17, S (total sample size) = 2098, *I*^2^ = 84.4%) and those reporting ≥2 falls in the past year (WAP = 59.0%; 95% CI = 40.0–76.0%, n = 17, S = 2078, *I*^2^ = 84.3%), (Table S6 in the **Online Supplementary Document**).

In residential home setting, data were only available for Egypt (Figure S6 and Table S8 in the **Online Supplementary Document**). The pooled prevalence of older adults reporting ≥1 fall in the past year was 57.3% (95% CI = 33.0–78.5%, n = 9, S = 662, *I*^2^ = 78.1%). We observed no significant difference between sexes and age groups. Additionally, in Egypt, we observed higher, but not statistically significant pooled prevalence among the older people in residential homes (previously mentioned), than among the general population (pooled prevalence = 40.8%; 95% CI = 32.3–49.8%, n = 5, S = 2172, *I*^2^ = 89.5%), or outpatients (pooled prevalence = 44.4%; 95% CI = 30.5–59.3%, n = 6, S = 1053, *I*^2^ = 95.0%, *P* = 0.462).

In health care setting, 60.1% of older trauma unit patients had a fall as the cause of their trauma (95% CI = 42.2–75.7%, n = 14, S = 2626, *I*^2^ = 94.0%) (Figure S5 in the **Online Supplementary Document**) and 49.3% of the older outpatients reported having ≥1 fall in the past year (95% CI = 33.9–64.8%, n = 13, S = 2242, *I*^2^ = 93%) (Figure S6 in the **Online Supplementary Document**). Regarding the nature of these falls, the weighted average proportion occurring at ground level accounted for 65.0% (95% CI = 56.3–72.9%, n = 5, S = 556, *I*^2^ = 82.8%) while falls from a height comprised 3.5% (95% CI = 0.9–12.8%, n = 6; S = 603, *I*^2^ = 92.8%) (Figure S7 in the **Online Supplementary Document**).

The results of our meta-regression are summarised in the appendix (Table S8 in the **Online Supplementary Document**). The univariable analysis indicated that the between-studies heterogeneity in prevalence was significantly influenced by population type, World Bank income levels, sample size, and sampling methodology. The multivariable model found that only population type and World Bank income levels are associated with the prevalence of falls among the older population. The meta-regression revealed that trauma unit patients (n = 14) had 288.0% higher odds (OR = 2.9; 95% CI = 1.1–7.2) of reporting falls compared to the community-dwelling population (n = 28). Additionally, we found that the pooled prevalence in LICs (n = 1) was 94.0% lower (OR = 0.06; 95% CI = 0.005–0.7) than in HICs (n = 29). We did not identify any evidence of temporal variation in prevalence (*P* = 0.261 for the year of data collection).

Predominantly, falls occurred at home (WAP = 66.1%), substantially in the bathroom, stairs, bedroom, and the kitchen (**Table 2**). A smaller proportion of falls, 22.9%, took place outdoors, with 10.1% being at work and 6.0% being in a health care setting.

**Table 2:** Weighted average proportions of older fallers by location of fall in the Middle East and North Africa

Location	Number of studies	Sample size	Number of fallers	Prevalence range (%)	Pooled estimate* (95% CI)	Heterogeneity measures	
						**Q test *P*-value**	***I*^2^ (%)**
**At policy level**							
Street	6	1546	137	8.2–10.1	8.9 (7.5–10.4)	0.98	0
Parking space	1	318	4	0.0–1.3	1.3 (0.3–3.2)	NA	NA
Traffic	1	253	33	0.0–13.0	13.0 (9.2–17.8)	NA	NA
**At institutional level**							
Hospital	8	1323	95	1.6–22.9	6.0 (2.5–13.8)	<0.01	90.3
**At community level**							
Occupational falls	4	3551	501	2.4–75.1	10.1 (1.6–44.2)	<0.01	99.7
Prayer place	3	993	46	2.5–5.9	4.1 (2.4–6.8)	0.07	63.0
Mall	1	590	26	0.0–4.4	4.4 (2.9–6.4)	NA	NA
Nursing homes	1	253	1	0.0–0.4	0.4 (0.0–2.2)	NA	NA
**At interpersonal level**							
Someone else’s home	2	843	81	7.5–10.5	9.6 (7.8–11.8)	0.18	44.9
Recreational centre	2	367	12	1.4–4.4	3.2 (1.6–6.5)	0.15	52.3
**At individual level**							
Home†	14	4630	1989	15.0–99.2	66.1 (46.6–81.3)	<0.01	98.8
Stairs	13	2223	492	4.0–100.0	27.3 (13.4–47.7)	<0.01	93.6
Bathroom	12	2111	725	19.0–74.0	34.5 (26.4–43.7)	<0.01	90.7
Bedroom	9	1490	260	7.0–27.0	17.0 (14.0–20.3)	0.01	59.8
Kitchen	5	1291	121	0.8–19.3	7.7 (3.3–17.1)	<0.01	83.0
Ladder	2	53	3	5.3–6.7	5.7 (1.8–16.1)	0.84	0.0
Front doorstep	2	675	179	12.9–28.5	20.9 (11.6–34.5)	<0.01	88.4
Way from bed to bathroom	2	53	20	26.7–42.1	37.7 (25.8–51.4)	0.30	6.6
Living room	1	119	10	0.0–8.4	8.4 (4.1–14.9)	NA	NA
Outdoor	10	1760	465	3.8–59.5	22.9 (14.2–34.7)	<0.01	95.0
Garden	2	372	50	6.3–28.6	14.1 (4.5–36.2)	<0.01	96.6
Other/unknown locations	7	2024	256	2.5–31.9	5.9 (2.9–11.6)	<0.01	97.1

CI – confidence interval, NA – not applicable

*Weighted average proportions were estimated using random-effect model meta-analysis. Weighted average proportions (*e.g*. at community level: street, parking space, and traffic) do not add up to 100% as each weighted average proportion derives from a different set of studies (depending on the available data).

†Home – community-dwelling setting or residential care facilities.

**Table 3** details the WAP of older adults reporting negative health consequences and hospital utilisation after a fall. The most common consequence was injury (WAP = 79.6%), 45.6% of older adults who experienced a fall visited a doctor or a hospital, 5.1% were admitted to an intensive care unit, and the death rate in the hospital following a fall was 7.5%. On average, 17.3% of older adults who experienced a fall reported suffering from anxiety or depressive symptoms afterward. Negative health-related consequences reported by individual studies are detailed in appendix (Table S9 in the **Online Supplementary Document**).

**Table 3:** Weighted average proportions of older fallers by health consequence and hospital utilisation after the fall in the Middle East and North Africa

Consequences	Number of studies	Sample size of fallers	Nb of fallers reporting that consequences	Prevalence range (%)	Pooled estimate* (95% CI)	Heterogeneity measures	
						**Q test *P*-value**	***I*^2^**(%)
**At institutional level**							
Healthcare utilisation†	3	1824	874	35.3–50.8	45.6 (37.8–29.8)	<0.01	98.4
ICU admissions	3	624	32	4.3–5.9	5.1 (3.6–7.2)	0.75	0.0
Surgeries	2	532	227	7.9–74.2	33.2 (4.1–85.2)	<0.01	99.4
**At individual level**							
Death	5	982	209	1.1–64.5	7.5 (1.5–29.8)	<0.01	98.2
Anxiety, depression, or depressive symptom	3	694	141	10.0–36.9	17.3 (8.0–33.2)	<0.01	0.0
Back pain	2	497	218	15.8–73.0	41.6 (10.0–82.0)	<0.01	99.3
Pain	2	288	198	59.0–87.0	75.2 (50.7–89.9)	<0.01	95.0
Difficulty in getting up alone	2	329	252	74.2–81.0	76.6 (71.7–80.9)	0.16	48.9
Affected walk	2	690	357	45.9–86.0	69.0 (35.7–89.9)	<0.01	98.0
Fracture	14	3175	1379	7.6–89.1	36.8 (21.9–54.8)	<0.01	98.6
Fracture of lower extremities	5	1821	525	6.6–52.2	25.4 (11.8–46.5)	<0.01	98.6
Fracture of trunk and chest	4	1882	157	2.9–13.0	6.9 (3.9–11.9)	<0.01	92.5
Fracture of upper extremities	3	1629	155	3.6–13.8	8.2 (4.2–15.4)	<0.01	94.4
Fracture of hip	3	961	159	3.6–47.0	12.1(2.8–40.1)	<0.01	98.9
Joint dislocation	4	570	80	5.4–27.1	12.7 (6.6–23.1)	<0.01	87.0
Fracture or dislocation	2	329	159	34.5–55.9	45.4 (31.1–60.5)	<0.01	92.6
Fracture of head and neck	2	1039	17	1.1–3.2	1.8 (0.8–3.8)	0.02	81.5
Fracture other unknown	2	869	51	0.3–17.6	2.6 (0.1–35.2)	<0.01	96.9
Bruises	10	2074	809	1.5–73.9	29.3 (15.2–49.0)	<0.01	96.4
Cut, laceration, abrasion, contusion	15	2596	493	5.9–60.9	21.8 (15.4–29.9)	<0.01	94.0
Injuries	7	1549	1084	22.5–99.6	79.6 (51.5–93.5)	<0.01	97.1
Injury of head	7	1184	228	1.6–61.2	11.9 (4.5–27.6)	<0.01	96.5
Injury of chest	3	492	114	12.0–27.9	18.8 (11.7–28.6)	<0.01	83.4
Injury of spine	3	492	78	9.8–18.4	14.4 (9.9–20.7)	0.09	58.0
Injury of abdomen	2	418	26	1.1–7.7	3.8 (0.9–14.3)	0.05	74.2
Injury of face	2	166	6	3.3–4.1	3.6 (1.6–7.8)	0.79	0.0
Injury of upper extremity	2	166	19	10.8–12.0	11.4 (7.4–17.2)	0.82	0.0
Injury of lower extremity	2	166	101	60.8–60.9	60.8 (53.2–68.0)	0.99	0.0
Injuries of soft tissue	2	276	9	2.2–3.8	3.3 (1.7–6.1)	0.48	0.0
No injury	3	232	84	30.2–47.6	36.9 (28.8–45.9)	0.07	62.3
Hematoma	2	193	76	34.6–49.2	40.4 (30.8–50.8)	0.05	73.3
Other consequence	2	724	45	3.0–6.9	6.2 (4.7–8.2)	0.10	63.9

CI – confidence interval, NA – not applicable

*Weighted average proportions were estimated using random-effect model meta-analysis. Weighted average proportions (*e.g*. at institutional level: hospital or doctor visit, ICU admissions, and surgeries) do not add up to 100% as each weighted average proportion derives from a different set of studies (depending on the available data).

†Healthcare utilisation – hospital or doctor visit.

**Figure 2:**
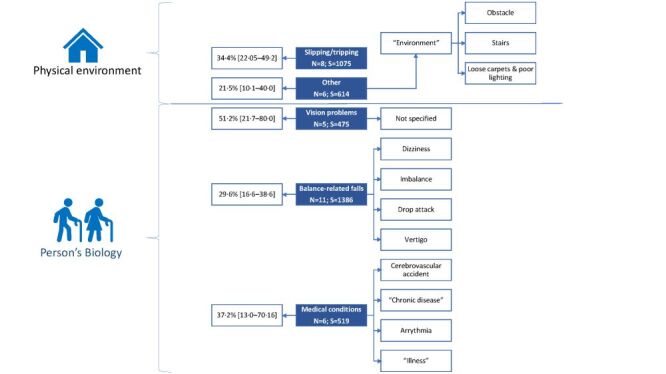
Weighted average proportions of older adult fallers by self-reported perceived reasons of fall in the Middle East and North Africa. Self-reported perceived reasons of fall reported based on the WHO system approach [2]. Weighted average proportions were estimated using random-effect model meta-analysis. N – number of studies, S – total simple size.

The self-reported reasons of falls perceived by the older adults when having a fall were mainly either related to the physical environment or to a person’s biology (**Figure 2**; Table S10 in the **Online Supplementary Document**). Medical conditions (WAP = 37.2%) and slipping/tripping (WAP = 34.4%) were the two most common self-reported perceived reasons of fall.

We detected publication bias (LFK index = 7.3) (Figure S8 in the **Online Supplementary Document**), suggesting a tendency for studies for reporting higher prevalences of falls to be more frequently published. This bias likely impacted the meta-analysis-derived pooled prevalence. Acknowledging publication bias, we calculated prediction intervals to provide more accurate insight into the true estimates (Figure S2–S7 in the **Online Supplementary Document**).

The identified publication bias, absence of studies in some countries, and scarcity of nationwide or registry-based studies may limit the external validity of our regional pooled prevalence estimates. To provide a more accurate estimate of the regional prevalence of fallers among the older population, we calculated a population-size weighted proportion for the MENA region (**Table 1**). Despite potential constraints in external validity, our pooled prevalence estimates demonstrate robustness, which are attributed to the strong internal validity of the included studies characterised by a low risk of measurement and analysis biases. However, heterogeneity between-studies likely affected the precision of our estimates. Consequently, we rated the certainty of the available evidence as moderate.

## DISCUSSION

Our study indicates that 17.6% of older adults in the MENA region report experiencing at least one fall, affecting 10 million individuals. While this prevalence is lower than global averages reported by the WHO (28–35%) [37], the USA (27.6%) [38], and the Latin America and Caribbean region (22–34%) [39], the health consequences associated with falls are substantial. The region records higher rates of fractures than the WHO’s global estimate [2], mental health issues like anxiety and depression, and a concerning 7.5% mortality rate – higher than the 1.3% in the USA [40].

The lower prevalence older fallers in low-income MENA countries (*i.e*. Sudan, Syria, and Yemen), may underestimate the regional population-size weighted prevalence, but this impact is likely minimal given that 90% of the population-size-weight comes from 17 other MENA countries and given that Sudan's low prevalence was estimated by its National Household Health Survey through multistage cluster random sampling [36]. Fall prevalence likely varies by income level, with lower rates in LICs due to limited data, underreporting, and reduced health care-seeking behaviour influenced by factors like inaccessible health care and war-related instability [41].

Our findings indicate that most falls happen at home, particularly in areas such as bathrooms, stairs, and kitchens. Additionally, significant numbers of falls occur outdoors, especially at work and in health care settings. These findings align with global trends [37,42] and emphasise the necessity for comprehensive, multifactorial interventions that are customised to individual profiles.

Falls among older adults in MENA not only result in physical injuries but also have mental health implications, with one-fifth of fallers experiencing anxiety or depression, an addition to the findings in the latest WHO report [2]. The substantial proportion of post-fall anxiety and depression highlights the need for comprehensive fall prevention strategies that address not only physical safety but also psychological support, as mental health consequences could lead to fear of falling, social isolation, and reduced physical activity, potentially creating a cycle of increased fall risk [43].This underscores the need for comprehensive fall prevention strategies that address both the physical and psychological aspects.

The influence of cultural and socioeconomic factors on falls in MENA requires consideration. Traditional family structures, where multiple generations commonly live together, may affect both fall occurrence and reporting patterns. The marked difference in prevalence between HICs (26.9%) and LICs (1.6%) likely reflects disparities in health care access and reporting systems rather than true prevalence differences. Limited health care facilities, particularly in countries experiencing instability, can affect both fall documentation and care-seeking behaviour. Our finding that only 45.6% of fallers sought medical care might reflect either limited health care access or traditional practices of family-based care. These contextual factors present unique challenges for implementing fall prevention strategies in MENA, where interventions must address both health care system constraints and cultural care practices.

The prevalence of fallers in residential homes was higher than in the community-dwelling setting, aligning with global observations [2,44]. We identified data for residential homes only in Egypt, possibly reflecting the persistence of traditional values in the region. Due to these traditional values, older adults in such countries often resist to relocate from home to residential care facilities [2]. This higher prevalence likely stems from greater frailty, comorbidities, mobility limitations, and cognitive impairments among residents, as well as environmental factors such as shared spaces and limited physical activity [5]. Effective fall management in residential homes should not only recognise all older adults at a high risk for falling but also consider traditional values, socioeconomic factors, frequent mobility assessments, environmental modifications, and staff training on fall prevention.

Our meta-analysis found no statistically significant sex-specific differences in fall prevalence. However, we acknowledge that the absence of statistical significance does not necessarily mean absence of differences between sexes particularly with global trends suggesting a higher fall rate among females [37]. Our subgroup analyses, including sex-stratified analyses, were conducted to investigate sources of heterogeneity rather than to infer causal relationships.

Our findings indicate that older adults primarily attribute the perceived reasons of falling to biological factors and the physical environment. Our findings highlight the importance of using multifactorial falls risk assessments, including disease history and environmental risk, as recommended by new guidelines [5,45]. Existing clinical guidelines in MENA provide a framework for health care staff to implement prevention strategies effectively and establishing safer environments for older adults across various settings [46,47]. Our comprehensive synthesis emphasises the necessity for integrated fall prevention strategies that go beyond individual-level interventions to address hazards in homes, communities, hospitals, and workplaces. Additionally, culturally appropriate, region-specific programmes should be designed to incorporate local values and promote interventions that respect traditional norms, focusing on maintaining safety and independence within the home environment.

Our work is the first to estimate the prevalence of fallers among the older population in MENA across low-, middle-, and high-income countries employing both meta-analysis and population-size weighted average methods. This dual approach allowed us to better account for variations in population sizes across countries. Specifically, while we included 52 studies from lower-middle-income countries (63.6% of older population), 34 from HICs (5.4%), three from upper-middle-income countries (6.2%), and one from LICs (9.8%), our population-weighted approach weighted each country's prevalence according to its older population size rather than number of studies. This prevented over-representation of income levels with more available data, resulting in more reliable and representative prevalence estimates for the region. Additionally, our review encompasses 90 articles on falls among older adults in MENA including 36 studies from the countries of the Cooperation Council, providing more comprehensive insights than the current research [9,10].

Our review identifies limitations, primarily stemming from the quantity and quality of available data by country. One challenge is the need for more granular data on falls in MENA. The scarcity of data for specific countries hampers accurate estimation of regional prevalence and addressing variations in health outcomes [2]. Particularly problematic is the absence of data from countries, such as Syria, Yemen, Algeria, Djibouti, Libya, and Oman, which collectively account for 14.3% of the region's older population. This lack of data presents a challenge in assessing falls related morbidity and mortality.

Additionally, a substantial proportion of the included primary studies had a risk of non-response and selection biases, which are common in cross-sectional studies using questionnaires [48]. To mitigate potential recall bias in fall reporting, we stratified our analyses by timeframe of fall occurrence, distinguishing between current falls documented in hospital records, falls within the past year, and lifetime history of falls, which allowed us to account for varying levels of recall reliability in our prevalence estimates.

The high level of heterogeneity in our meta-analyses, common when pooling prevalence rates, suggests some inconsistency in the prevalence measures [49]. This heterogeneity may result from variations in study design, timing, setting, and differences in sample sizes, which can lead to minimal overlap of CIs [49]. While we used the WHO definition of falls in our review, only 42.2% of included studies provided clear fall definitions, which may have introduced variability in how fall events were captured across studies. We addressed this limitation by providing prediction intervals alongside our pooled estimates and considering it in our certainty of evidence assessment. Additional sources of heterogeneity may include cultural and environmental differences and health care access and reporting practices. These unmeasured sources of heterogeneity could particularly affect our estimates in LICs where limited health care access may lead to underreporting, suggesting our pooled estimates might be conservative in these settings. To address these limitations, we conducted sub-group analysis and meta-regression to explore the sources of heterogeneity and improve the representativeness of our findings. Despite these efforts, the calculated pooled prevalence should be considered a weighted average rather than a strict estimation of mean prevalence for the considered outcomes and subpopulations. These weighted averages reflect the diversity of fall reporting and health care practices across the region rather than a uniform regional estimate. We also detected a publication bias, which we addressed by computing the 95% prediction interval of the corresponding pooled prevalence. Given the detected publication bias, the prediction intervals we report alongside our pooled estimates provide a more conservative range for interpreting the true prevalence of falls in the MENA region, as they account for both between-study heterogeneity and potential publication bias.

## CONCLUSIONS

The MENA prevalence of older adults reporting at least one fall is 17.6%, affecting 10 million individuals. Our study reveals substantial consequences from falls, including fractures, mental health issues, and a 7.5% death rate in the hospital post-fall. The residential home remains a critical area for fall prevention, as seen globally. Self-reported reasons for falls highlight biological factors (*e.g*. balance-related factors and medical conditions) and physical environment factors (*e.g*. slipping and tripping). Our synthesis underscores the need for granular, region-specific data to enhance regional understanding of fall epidemiology, inform multifactorial prevention strategies and guide public health policy in MENA.

## Additional material


Online Supplementary Document
