# Supplementary material for: The epidemiology of unintentional falls among older people in the Middle East and North Africa: a systematic review and meta-analysis
Source: J Glob Health. 2025 Mar 14;15:04072. doi: 10.7189/jogh.15.04072 (PMC11907375; doi:10.7189/jogh.15.04072)
Supplement: Online Supplementary Document [file jogh-15-04072-s001.pdf]

Supplement to: Chaabna K, Jithesh A, Khawaja S, Aboughanem J, Mamtani R, Cheema S. The epidemiology of unintentional falls among older people in the Middle East and North Africa: a systematic review and meta-analysis. J Glob Health. 2025;15:04072.

Table S1: Prisma 2020 checklist

Section and Topic	Item #	Checklist item	Location where item is reported
**TITLE**			
Title	1	Identify the report as a systematic review.	Page 1
**ABSTRACT**			
Abstract	2	See the PRISMA 2020 for Abstracts checklist.	Page 2
**INTRODUCTION**			
Rationale	3	Describe the rationale for the review in the context of existing knowledge.	Page 3
Objectives	4	Provide an explicit statement of the objective(s) or question(s) the review addresses.	Page 3
**METHODS**			
Eligibility criteria	5	Specify the inclusion and exclusion criteria for the review and how studies were grouped for the syntheses.	Pages 4-5
Information sources	6	Specify all databases, registers, websites, organisations, reference lists and other sources searched or consulted to identify studies. Specify the date when each source was last searched or consulted.	Page 5
Search strategy	7	Present the full search strategies for all databases, registers and websites, including any filters and limits used.	Page 5 & Boxes S1-2
Selection process	8	Specify the methods used to decide whether a study met the inclusion criteria of the review, including how many reviewers screened each record and each report retrieved, whether they worked independently, and if applicable, details of automation tools used in the process.	Page 5
Data collection process	9	Specify the methods used to collect data from reports, including how many reviewers collected data from each report, whether they worked independently, any processes for obtaining or confirming data from study investigators, and if applicable, details of automation tools used in the process.	Page 5
Data items	10a	List and define all outcomes for which data were sought. Specify whether all results that were compatible with each outcome domain in each study were sought (e.g. for all measures, time points, analyses), and if not, the methods used to decide which results to collect.	Page 4
	10b	List and define all other variables for which data were sought (e.g. participant and intervention characteristics, funding sources). Describe any assumptions made about any missing or unclear information.	Pages 4-6
Study risk of bias assessment	11	Specify the methods used to assess risk of bias in the included studies, including details of the tool(s) used, how many reviewers assessed each study and whether they worked independently, and if applicable, details of automation tools used in the process.	Pages 5-6
Effect measures	12	Specify for each outcome the effect measure(s) (e.g. risk ratio, mean difference) used in the synthesis or presentation of results.	Pages 5-6
Synthesis methods	13a	Describe the processes used to decide which studies were eligible for each synthesis (e.g. tabulating the study intervention characteristics and comparing against the planned groups for each synthesis (item #5)).	Pages 5-6
	13b	Describe any methods required to prepare the data for presentation or synthesis, such as handling of missing	Pages 5-6

Section and Topic	Item #	Checklist item	Location where item is reported
		summary statistics, or data conversions.	
	13c	Describe any methods used to tabulate or visually display results of individual studies and syntheses.	Pages 5-6
	13d	Describe any methods used to synthesize results and provide a rationale for the choice(s). If meta-analysis was performed, describe the model(s), method(s) to identify the presence and extent of statistical heterogeneity, and software package(s) used.	Pages 5-6
	13e	Describe any methods used to explore possible causes of heterogeneity among study results (e.g. subgroup analysis, meta-regression).	Pages 5-6
	13f	Describe any sensitivity analyses conducted to assess robustness of the synthesized results.	Pages 5-6
Reporting bias assessment	14	Describe any methods used to assess risk of bias due to missing results in a synthesis (arising from reporting biases).	Pages 5-6
Certainty assessment	15	Describe any methods used to assess certainty (or confidence) in the body of evidence for an outcome.	Pages 5-6
**RESULTS**			
Study selection	16a	Describe the results of the search and selection process, from the number of records identified in the search to the number of studies included in the review, ideally using a flow diagram.	Page 6
	16b	Cite studies that might appear to meet the inclusion criteria, but which were excluded, and explain why they were excluded.	Box S3
Study characteristics	17	Cite each included study and present its characteristics.	Table S2
Risk of bias in studies	18	Present assessments of risk of bias for each included study.	Table S3 and Figure S1
Results of individual studies	19	For all outcomes, present, for each study: (a) summary statistics for each group (where appropriate) and (b) an effect estimate and its precision (e.g. confidence/credible interval), ideally using structured tables or plots.	Table S2
Results of syntheses	20a	For each synthesis, briefly summarise the characteristics and risk of bias among contributing studies.	Page 6
	20b	Present results of all statistical syntheses conducted. If meta-analysis was done, present for each the summary estimate and its precision (e.g. confidence/credible interval) and measures of statistical heterogeneity. If comparing groups, describe the direction of the effect.	Pages 7-8
	20c	Present results of all investigations of possible causes of heterogeneity among study results.	Pages 7-8
	20d	Present results of all sensitivity analyses conducted to assess the robustness of the synthesized results.	Pages 7-8
Reporting biases	21	Present assessments of risk of bias due to missing results (arising from reporting biases) for each synthesis assessed.	Pages 7-8
Certainty of	22	Present assessments of certainty (or confidence) in the body of evidence for each outcome assessed.	Page 8

Section and Topic	Item #	Checklist item	Location where item is reported
evidence			
**DISCUSSION**			
Discussion	23a	Provide a general interpretation of the results in the context of other evidence.	
	23b	Discuss any limitations of the evidence included in the review.	
	23c	Discuss any limitations of the review processes used.	
	23d	Discuss implications of the results for practice, policy, and future research.	
**OTHER INFORMATION**			
Registration and protocol	24a	Provide registration information for the review, including register name and registration number, or state that the review was not registered.	Page 8
	24b	Indicate where the review protocol can be accessed, or state that a protocol was not prepared.	Page 4
	24c	Describe and explain any amendments to information provided at registration or in the protocol.	Na
Support	25	Describe sources of financial or non-financial support for the review, and the role of the funders or sponsors in the review.	Page 10
Competing interests	26	Declare any competing interests of review authors.	Page 10
Availability of data, code and other materials	27	Report which of the following are publicly available and where they can be found: template data collection forms; data extracted from included studies; data used for all analyses; analytic code; any other materials used in the review.	Supplementary material

Box S1: Search Strategy—Main search

Electronic databases	Date of search
**PubMed/Medline: 815**	**2nd November 2021**

("aged"[MeSH Terms] OR "aged"[TEXT WORD] OR "elderly*"[TEXT WORD] OR "older population*"[TEXT WORD] OR geriatric*[TEXT WORD] or “older adult*”[TEXT WORD] OR "Falls in older adult*"[TEXT WORD])
AND
("accidental falls"[MeSH Terms] OR ("accidental"[TEXT WORD] AND "falls"[TEXT WORD]) OR "accidental falls"[TEXT WORD] OR "falling"[TEXT WORD] OR "falls"[TEXT WORD] OR "fallings"[TEXT WORD] or "accidental slip*"[TEXT WORD] OR “unintentional fall*”[TEXT WORD] or “loss of balance”[TEXT WORD])
AND
("Qatar"[Mesh] OR "Bahrain"[Mesh] OR "Oman"[Mesh] OR "Saudi Arabia"[Mesh] OR "Kuwait"[Mesh] OR "United Arab Emirates"[Mesh] OR "Yemen"[Mesh] OR "Egypt"[Mesh] OR "Jordan"[Mesh] OR "Lebanon"[Mesh] OR "Syria"[Mesh] OR "Iraq"[Mesh] OR "Algeria"[Mesh] OR "Libya"[Mesh] OR "Morocco"[Mesh] OR "Tunisia"[Mesh] OR "Djibouti"[Mesh] OR "Sudan"[Mesh] OR "South Sudan"[Mesh] OR "Pakistan"[Mesh] OR "Africa, Northern"[Mesh] OR "Africa, Eastern"[Mesh] OR "middle east"[Mesh] OR "Arabs"[Mesh] OR "UAE"[Title/Abstract] OR "U.A.E"[Title/Abstract] OR "Emirat*"[Title/Abstract] OR "United Arab Emirates"[Title/Abstract] OR "Qatar*"[Title/Abstract] OR "Oman*"[Title/Abstract] OR "Saudi Arabia*"[Title/Abstract] OR "Saudi*"[Title/Abstract] OR "Kuwait*"[Title/Abstract] OR "Bahrain*"[Title/Abstract] OR "Yemen*"[Title/Abstract] OR "Egypt*"[Title/Abstract] OR "Jordan*"[Title/Abstract] OR "Leban*"[Title/Abstract] OR "Syria*"[Title/Abstract] OR "Iraq*"[Title/Abstract] OR "West Bank*"[Title/Abstract] OR "Gaza*"[Title/Abstract] OR "Palestin*"[Title/Abstract] OR "Algeria*"[Title/Abstract] OR "Libya*"[Title/Abstract] OR "Morocc*"[Title/Abstract] OR "Tunis*"[Title/Abstract] OR "Djibouti*"[Title/Abstract] OR "Sudan*"[Title/Abstract] OR "South Sudan*"[Title/Abstract] OR "Pakistan*"[Title/Abstract] OR "North Africa*"[Title/Abstract] OR "North-Africa*"[Title/Abstract] OR ("Africa"[Title/Abstract] AND "Northern"[Title/Abstract]) OR "Northern Africa"[Title/Abstract] OR "East Africa"[Title/Abstract] OR ("Africa"[Title/Abstract] AND "Eastern"[Title/Abstract]) OR "Maghreb"[Title/Abstract] OR "Maghrib"[Title/Abstract] OR "Arab*"[Title/Abstract] OR "Bedouin*"[Title/Abstract] OR "Gulf Cooperation Council"[Title/Abstract] OR "GCC"[Title/Abstract] OR "Middle East"[Title/Abstract] or “MENA”[Title/Abstract] OR "Middle East and North Africa"[Title/Abstract])

**Web of Science: 814**	**2nd November 2021**

(elderly OR (older population) OR (older adults) OR geriatric)
AND
((accidental falls) OR falling OR falls OR (accidental slip) OR (unintentional fall) OR (loss of balance))
AND
(Qatar OR Bahrain OR Oman OR (Saudi Arabia) OR Kuwait OR (United Arab Emirates) OR Yemen OR Egypt OR Jordan OR Lebanon OR Syria OR Iraq OR Algeria OR Libya OR Morocco OR Tunisia OR Djibouti OR Sudan OR South Sudan OR Pakistan OR Palestine OR "middle east" OR "Arabs" OR UAE OR U.A.E OR Emirat* OR (Gulf Cooperation Council) OR GCC OR MENA OR (Middle East and North Africa)

Box S2: Search Strategy—Supplementary search

**Search engine**	Date
**Google Scholar**	
Search 1a:	29th November 2022-5th May 2024
With **all** of the words: ~**Accidental falls**<br>Exact phrase: Elderly<br>With **at least one** of the words: MENA "Middle East" "North Africa" Algeria Bahrain Djibouti Egypt Iraq Jordan Kuwait Lebanon Libya Morocco Oman	
Search 1b:	29th November 2022-5th May 2024
With **all** of the words: ~**Accidental falls**<br>Exact phrase: Elderly<br>With **at least one** of the words: GCC “Gulf Cooperation Council” Maghreb Pakistan Palestine Qatar “Saudi Arabia” KSA Sudan Syria Tunisia “United Arab Emirates” UAE Yemen	
Search 2a:	30th November 2022-5th May 2024
With **all** of the words: ~**Fear of falling**<br>Exact phrase: Elderly<br>With **at least one** of the words: MENA "Middle East" "North Africa" Algeria Bahrain Djibouti Egypt Iraq Jordan Kuwait Lebanon Libya Morocco Oman	
Search 2b:	30th November 2022-5th May 2024
With **all** of the words: ~**Fear of falling**<br>Exact phrase: Elderly<br>With **at least one** of the words: GCC “Gulf Cooperation Council” Maghreb Pakistan Palestine Qatar “Saudi Arabia” KSA Sudan Syria Tunisia “United Arab Emirates” UAE Yemen	
Search 3a:	4th December 2022-5th May 2024
With **all** of the words: ~ **Elderly**<br>Exact phrase: Risk of falls<br>With **at least one** of the words: MENA "Middle East" "North Africa" Algeria Bahrain Djibouti Egypt Iraq Jordan Kuwait Lebanon Libya Morocco Oman	
Search 3b:	4th December 2022-5th May 2024
With **all** of the words: ~ **Elderly**<br>Exact phrase: Risk of falls<br>With **at least one** of the words: GCC “Gulf Cooperation Council” Maghreb Pakistan Palestine Qatar “Saudi Arabia” KSA Sudan Syria Tunisia “United Arab Emirates” UAE Yemen	
Search 4a:	19th July 2023-5th May 2024
With **all** of the words: ~ Elderly, ~Accidental, ~Fear of falling<br>Exact phrase: Risk of falling, Risk of falls, Elderly<br>With **at least one** of the words: GCC “Gulf Cooperation Council” Maghreb Pakistan Palestine Qatar “Saudi Arabia” KSA Sudan Syria Tunisia “United Arab Emirates” UAE Yemen	
Search in Arabic	30th October 2023
"سقوط" AND "كبار السن" AND "سوريا"8<br>"سقوط" AND "كبار السن" AND "سودان"1<br>"سقوط" AND "كبار السن" AND "اليمن"6<br>"سقوط" AND "كبار السن " AND "ليبيا"3	

Table S2: Characteristics of included studies

Reference	Country	Response rate %	Study Design	Sampling	Year of data collection	Description	Mean Age (SD), Range	Study sample size	Sex n (%)
HIGH-INCOME COUNTRIES									
Ibrahim, 2021 [1]	Kuwait	100	National registry-based retrospective	All target population	2016	• All patients with **accidental falls** admitted to all public hospitals in Kuwait.<br>• National database of inpatient hospitalizations maintained by the National Center for Health Information, Ministry of Health.	≥65	760	NR*
Al-Balushi, 2012 [2]	Oman	NA	CS	Convenience	2010	• All patients who attended Al Moabelah Health Center in the Muscat governorate and presented with **injury.**	≥60	72	M: 46 (63.8)<br>F: 26 (36.2)
Abdelrahman, 2018 [3]	Qatar	100	National registry-based retrospective	All target population	2008–2011	• Patients with a **history of a fall at home** treated at the Hamad Trauma Center (the National Level I trauma center), Trauma Surgery Section, Department of Surgery, Hamad General Hospital.<br>Data from the trauma national registry.	≥60	35	NR*
Almawlawi, 2011 [4]	Qatar	NR	CS	Random	2008	• Elderly patients attending 12 out of the 24 Primary Health Centers.	≥60	355	M: 165 (46.5)<br>F: 190 (53.5)
Alyazeedi, 2019 [5]	Qatar	100	National registry-based retrospective	All target population	2016–2018	• All older adults visiting geriatrics **outpatient** clinics at Rumailah Hospital, Hamad Medical Corporation, the main geriatric hospital in the country.<br>Data collected from the national registry.	72.8 (6.9)	3537	M: 1947 (55)<br>F: 1590 (45)
Bener, 2011 [6]	Qatar	100	Retrospective	All target population	2001–2006	• Patient with **head and/or neck injuries due to falls** that were treated at the Accident and Emergency and eight other Trauma Centers of Hamad Medical Corporation.	≥60	134	NR*
Bener, 2012 [7]	Qatar	100	Retrospective	All target population	2006–2010	• Patients treated in the **accident and emergency** department of the Hamad General Hospital and eight other **Trauma** Centers of the Hamad Medical Corporation.	>60	1542	NR*
El-Matbouly, 2013 [8]	Qatar	100	Retrospective	All target population	2008–2011	• Patients with **traumatic brain injuries** (TBI) admitted to the Level I trauma Center at Hamad General Hospital.	61–80	47	M/F (Nb NR)
El-Menyar, 2013 [9]	Qatar	100	Retrospective	All target population	2008–2010	• All patients with **traumatic chest injury** (TCI) admitted to the Level I trauma center at Hamad Medical Center (HMC)	60–108	62	M: 53 (86.0)

						Data was collected from the Trauma Surgery at Hamad General Hospital.			F: 9 (14.0)
Hassan, 2023 [10]	Qatar	100	CS	All target population	2017–2022	• Elderly patients with at least one encounter with primary health care centers. Data was collected from all primary care centers in the country.	Median: 65.0, IQ: 62.0-70.0	68 194	M: 40 166 (58.9) F: 28 028 (41.1)
Mekkodathil, 2020 [11]	Qatar	100	Retrospective	All target population	2010–2017	• Patients admitted to the Hamad Trauma Center (HTC) of Hamad General Hospital (HGH) with **fall-related injuries.**	≥60	326	NR*
Bener, 2010 [12]	Qatar	100	Retrospective	All target population	2003–2007	• Elderly patients with mild, moderate, or severe **traumatic brain injuries** who were treated in the Accident and Emergency Department of Hamad General Hospital and from eight other Trauma Centers of Hamad Medical Corporation.	>65	72	NR*
Al Senany, 2015 [13]	Saudi Arabia	NR	CS	NR	2013**	• Elderly patients visiting a major hospital in Jeddah and who are able to participate in activities of daily living with or without ambulatory aid.	67.9 (7.7), 60–90	55	M/F (Nb NR)
Alabdullgader, 2021 [14]	Saudi Arabia	96.1	CS	NR	2019	• **Elderly patients** visiting 10 out of 19 randomly selected primary health centers in Unaizah City, Qassim Province.	70.3 (8.2) for males 68.6 (6.8) for females, 60–90	269	M: 133 (49.4) F: 136 (50.6)
Alawad, 2020 [15]	Saudi Arabia	100	Retrospective	All target population	2016–2018	• Patients with **traumatic spinal injuries** at the King Saud Medical City (KSMC) level 1 trauma center, which is the largest in the country with annual emergency department.	≥60	74	NR*
Alharbi, 2023 [16]	Saudi Arabia	NR	NR	Convenience	2021**	• **Ambulatory community-dwelling** individuals were recruited from two rehabilitation centers (general government and private sector hospitals).	≥65	114	M: 73 (64.1) F: 41 (35.9)
Aljawadi, 2018 [17]	Saudi Arabia	NA	National CS	Random	2006–2017	• Saudis who participated in the Saudi National Survey for Elderly Health (SNSEH).	≥60	2946	M: 1485 (50.4) F: 1461 (49.6)
Almegbel, 2017 [18]	Saudi Arabia	NR	CS	Convenience	2016	• Saudi elderly citizens living in Riyadh.	68.8 (9.0), 60–111	1182	M: 545 (46.1) F: 637 (53.9)
Al-Qahtani, 2020 [19]	Saudi Arabia	NA	CS	Random	2019	• Elderly Saudi nationals either coming for themselves or accompanying other patients to the health-care centers (PHCs) in Khamis Mushait.	70.6 (NR), 60–102	171	M: 171 (100.0)

Alqarni, 2021[20]	Saudi Arabia	100	CS	All target population	2015–2020	• Elderly patients with **hip fracture** who were admitted to the orthopedic department at King Abdulaziz Medical City in Jeddah.	79.6 (8.5), 65–88	130	M: 75 (57.7) F: 55 (42.3)
Alshammari, 2018 [21]	Saudi Arabia	NR	CS	Convenience	2015–2016	• Elderly patients who visited the largest mosques, malls, hospitals, and health centers in Riyadh, which was divided into five sections: North, South, Central, East, and West.	≥60	357	M: 168 (47.1) F: 189 (52.9)
Assiri, 2020 [22]	Saudi Arabia	NR	CS	Random	2018**	• Elderly adults who attended five randomly selected primary health care (PHC) centers in Abha City.	75.9 (10.3), 65–97	402	M: 206 (51.2) F: 196 (48.8)
Attar, 2021[23]	Saudi Arabia	NA	CS	Convenience	2015–2020	• Elderly who have been diagnosed with **falls** at the National Guard Hospital in Jeddah.	77.6 (8.1), 65–85	279	M: 149 (53.4) F: 130 (46.6)
El-Sobkey, 2011 [24]	Saudi Arabia	NA	Retrospective	Convenience	2009–2010	• Recruited Saudi community-dwelling older adults with a history of falls who were able to walk without human assistance and follow given instructions.	67.0 (7.3)	24	M: 9 (37.5) F: 15 (62.5)
Ullah, 2019 [25]	Saudi Arabia	100	Retrospective	All target population	2011–2013	• All patients with hemorrhagic or ischemic **stroke** admitted at the inpatient **rehabilitation unit** at King Fahad Medical City, Riyadh.	>65	48	NR*
Alenazi, 2023 [26]	Saudi Arabia	NR	CS	Convenience	2022	• **Community-dwelling** Saudi elderly from various regions across Saudi Arabia, including Riyadh, Alkharj, Jazan, Jeddah, Arar, Hail, and Tabuk, were recruited. Participants were sourced from the community, including locations such as mosques, malls, clinics, and other public areas	≥60	96	NR*
Adam, 2008 [27]	UAE	100	Registry-based prospective	All target population	2003–2006	• Patients with **injuries** who have stayed on surgical wards for more than 24h, died in emergency department, or died after admission.<br>Data of the Trauma Registry of Al-Ain Hospital.<br>This population is a subgroup of a wider population studied in Hefny, 2016.	69 (NR), 60–100	121	M: 70 (57.8) F: 51 (42.2)
Alao, 2021[28]	UAE	NR	Registry-based retrospective	NR	2003–2006	• Trauma patients who were hospitalized at Al-Ain Hospital for more than 24 hours or died after arrival.<br>Data were extracted from the Al-Ain Hospital trauma registry	Median: 74, 65–100	266	M: 133 (50.0) F: 133 (50.0)
Alzaabi, 2022 [29]	UAE	NR	CS	Convenience	2020–2021	• **Community-dwelling** elderly were recruited from community centers. Individuals with a history of smoking, chronic obstructive pulmonary disease, chronic cardiac disease, asthma, lower limb surgery (e.g., hip or knee arthroplasty), lower limb deformity requiring a heel lift on	75 (3.6)	100	M: 50 (50.0) F: 50 (50.0)

						their shoe, and visual or vestibular problems were excluded.			
Hefny, 2016 [30]	UAE	100	Registry-based retrospective	All target population	2003–2007	• All elderly **trauma patients** who were admitted to Al-Ain Hospital or died in the Emergency Department.<br>Note: Hospitalized trauma patients in Al-Ain city were mainly managed in two major hospitals (Al-Ain and Tawam Hospitals). 80% of them were treated at Al-Ain Hospital.<br>Data of the Trauma Registry of Al-Ain Hospital<br>**A subset group of this study was part of a previous study, which was published in Adam et al., 2008.**	72.2 (9.6)	154	M/F (Nb NR)
Sharif, 2018 [31]	UAE	72.6	CS	NR	2017	• Elderly individuals residing in Dubai and Sharjah.	≥60	370	M: 114 (30.8)<br>F: 256 (69.2)
Aleid, 2023 [32]	Saudi Arabia	NR	National CS	Purposive	2023	• Older adults presenting to **emergency** rooms in various provinces of the Saudi Arabia, encompassing Middle Eastern, Northern, South, and Western provinces.	≥65	21	NR
Alqurayshah, 2023[33]	Saudi Arabia	NR	CS	Purposive	2023	• Elderly individuals attending **primary healthcare** centers in Najran city, Saudi Arabia, were included in the study. Those with severe cognitive and communication impairments, which hindered their ability to recall fall incidents, and those with untreated mental illnesses, whose responses may be unreliable, were excluded.	≥65	377	M: 162 (43.0)<br>F: 215 (57.0)
Alshehri, 2024[34]	Saudi Arabia	NR	National CS	NR	2022	• Elderly individuals, both **infected and non-infected** with COVID-19, from the central, western, and eastern provinces of Saudi Arabia were included in the study. Subjects were excluded if they had neurological disorders, peripheral neuropathy, vestibular impairments, or had undergone arthroplasty. Additionally, those taking medications that could affect balance were also excluded.	60–75	86	NR
UPPER-MIDDLE INCOME COUNTRIES									
Amin, 2019 [35]	Iraq	NR	CS	NR	2014–2015	• Elderly **diabetic** patients **with a history of falls** at the Shar-hospital in Sulaimani City, Kurdistan.	77.7 (7.1), 65–90	150	M: 62 (41.0)<br>F: 88 (59.0)
Stewart, 2016 [36]	Iraq	NR	NR	Random	2014	• Individuals living in **households** in Baghdad.	≥65	228	NR*

Muhaidat, 2022 [37]	Jordan	NR	CS	Convenience	2020**	• **Community-dwelling** elderly individuals were recruited from shopping malls, local community centers, personal contacts, and the waiting rooms of **outpatient** clinics at the University of Jordan Hospital in the capital, Amman. Participants were excluded if they had any health condition affecting their ability to walk 10 meters or if they were unable to provide consent.	60–83	120	M: 39 (32.5) F: 81 (67.5)
LOWER-MIDDLE INCOME COUNTRIES									
Adly, 2020 [38]	Egypt	NR	CS	NR	2018	• Patients admitted to a geriatric **inpatient** department in Ain Shams University Hospital, Cairo.	68.7 (7.3)	190	M: 94 (49.5) F: 96 (50.5)
Al Tehewy, 2015 [39]	Egypt	NA	Observational longitudinal	Random	2009	• Elderly patients admitted to the **internal medicine** department at Ain Shams University Hospital.	67.6 (6.7), 60–90	411	M: 224 (54.5) F: 187 (45.5)
Aly, 2021 [40]	Egypt	100	Retrospective	All target population	2020	• Women with a history of **COVID-19** confirmed by PCR, and with at least one month elapsed since testing negative.	73.2 (6.4)	115	F: 115 (100.0)
Amer, 2018 [41]	Egypt	NR	CS	Convenience	2016**	• Elderly patients in N**ursing homes** in Ain Shams, Cairo AND Elderly dwelling in community in Cairo.	≥60	200	M/F (Nb NR)
El Sayed, 2023 [42]	Egypt	NR	CS	Convenience	2021**	• Elderly who are able to communicate, and living independently at home, in rented accommodation, in a hostel, or in a **retirement home**.	≥60	144	NR
El-Gilany, 2011 [43]	Egypt	82	CS	All target population	2010	• Elderly living in Menut Sandob village, Mansoura region.	≥60	766	M/F (Nb NR)
El-Gilany, 2013 [44]	Egypt	NR	Pre-Post Intervention	Convenience	2010–2011	• Elderly **with history of fall** within the last year living in Menyet Sandoub village, Mansoura region.	69.6 (6.2), 60–91	63	M: 21 (33.3) F: 42 (66.7)
El-Kawaly, 2016 [45]	Egypt	NR	CS	Convenience	2014–2015	• Elderly living at four different **nursing homes** in Cairo.	72.1 (8.8)	100	M: 49 (33.3) F: 51 (66.7)
El-Rahman, 2014 [46]	Egypt	NR	CS	NR	2013–2014	• Elderly living in Dar Al Mogama governmental elderly home in Damanhur, El-Behaira Governorate- Egypt. It is the only elderly home in the governorate.	68.8 (7.1), 60-84	114	M: 60 (52.6) F: 54 (47.4)

Elsamahy, 2019 [47]	Egypt	NR	CS	Convenience	2017–2018	• Elderly living in Grise village, which is affiliated to Ashmoon city in El- Monofya Governorate. Free from dementia.	≥60	272	M: 133 (48.9) F: 139 (51.1)
Hamed, 2017 [48]	Egypt	86.3	CS	Random	2015–2016	• Elderly population from the 6 districts of Sohag governorate.	≥60	1,034	M: 392 (37.9) F: 642 (62.1)
Ismail, 2018 [49]	Egypt	NR	Quasi-experimental	Convenience	2016–2017	• Elderly residents from 7 **geriatric homes** in different districts in Cairo.	69.3 (6.2)	120	M: 59 (49.2) F: 61 (50.8)
Kamel, 2013 [50]	Egypt	NR	CS	Convenience	2010–2011	• Elderly receiving medical services at the two urban **primary health care centers** in Suez city.	>60	340	M: 125 (36.8) F: 215 (64.2)
Khater, 2012 [51]	Egypt	100	Prospective	Convenience	2009–2010	• Elderly residents in **nursing homes** who exhibit both mobility and cognitive competence.	71.9 (7.2), 60–90	84	M: 36 (42.9) F: 48 (57.1)
Mabrouk, 2003 [52]	Egypt	NA	Prospective	Convenience	1995–2001	• Elderly patients with **burn injuries** who were admitted to the Ain Shams University burns unit.	64.5 (NR), 60–75	97	M: 44 (45.3) F: 53 (54.7)
Mahmoued, 2014 [53]	Egypt	NR	CS	Purposive	2012–2013	• Elderly residing at **home** in the rural area of Zagazig district, who are not bedridden or mentally impaired, and maintain independence in performing their daily activities.	60–80	236	M: 106 (44.9) F: 130 (55.1)
Makhlouf, 2000 [54]	Egypt	13 to 69	CS	Convenience	1998–1999	• Elderly individuals from six out of fourteen **geriatric institutions** in Alexandria were included in the study, with exclusion criteria applied to those with advanced cognitive impairment.	73.5 (7.5) for M 73.4 (7.7) for F	165	M: 62 (37.6) F: 103 (62.4)
Mohamed, 2019 [55]	Egypt	NR	Quasi-experimental	Purposive	2017–2018	• Elderly from three **geriatric homes** in minia city (Dar Omar Bn El khatab, Dar El Qedesa Hena, and Dar El raee el saleh) who are able to exercise and carry out daily activities.	67.3 (6.4), 60–85	80	M: 49 (61.3) F: 31 (38.7)
Mohammed, 2018 [56]	Egypt	NR	CS	Random	2014–2015	• Elderly individuals residing in areas of Sharkia Governorate and who were not bedridden or hospitalized.	70.7 (9.3), 60–98	408	M: 205 (50.2) F: 203 (49.8)
Mohammed, 2019 [57]	Egypt	100	Quasi-experimental	NR	2015–2016	• **Community-dwelling** elderly attending Fanara Family Medicine Center and were referred to the fall intervention program assessed at baseline.	65.1 (4.7), 60–79	100	M: 34 (34.0) F: 66 (66.0)

Mohammed, 2021 [58]	Egypt	NR	CS	Convenience	2019–2020	• Elderly residents living in one of three **geriatric homes** and **at the outpatient clinics of** Minia City-Minia Government, attending the diabetic, orthopedic, ophthalmic, and medical clinics.	66.1 (5.6)	384	M: 186 (48.4) F: 198 (51.6)
Mohsen, 2019 [59]	Egypt	NR	Quasi-experimental	Random	2017–2018	• Elderly individuals with a history of falls residing in two villages of Ghemreen village and Manshat Sultan village in Menoufia governorate.	≥60	130	M/F (Nb NR)
Sakr, 2006 [60]	Egypt	100	Retrospective	All target population	1991–2000	• All patients who had **mandibular fractures admitted** at the University Hospital of Alexandria.	≥60	173	NR*
Saleh, 2018 [61]	Egypt	NR	CS	Random	2017–2018	• Elderly individuals residing in both urban and rural areas affiliated with Mansoura city.<br>• In urban areas, participants were recruited from four health centers: El-Hawar Health Center, Sandowb Center, Toriel Center, and Gedela Center.<br>• In rural areas, participants were drawn from four villages: Badaway Village, Meet Badr Khamis Village, Meet Ali Village, and Nekita Village.<br>• Eligible participants in both urban and rural settings were those who were not bedridden or reliant on a wheelchair for mobility.	70.3 (7.0), 60–85	528	M: 236 (44.7) F: 292 (55.3)
Saudi, 2021[62]	Egypt	NR	CS	Convenience	2018–2019	• Elderly patients receiving care at the Family Medicine **Outpatient** Clinic affiliated with Suez Canal University Hospitals in Ismailia Governorate.	66.5 (4.9)	404	M: 189 (46.8) F: 215 (53.2)
Sayed, 2015 [63]	Egypt	NR	CS	Purposive	2015	• Elderly living in four different geriatric homes in East-Cairo.	≥60	119	M: 59 (49.6) F: 60 (50.4)
Sharaf, 2008 [64]	Egypt	100	CS	All target population	2006**	• Older adults recruited from all government **assisted living facilities (elderly homes)** in Alexandria and 2 Arabic speaking private facilities. They are independent or semi-independent and able to care for themselves, mobile with or without assistance, and free from any cognitive or physical impairment, psychiatric disease, or infection.	73.2 (8.9), 60–102	208	M: 83 (39.9) F: 125 (60.1)
Tawfik, 2021[65]	Egypt	100	Prospective	All target population	2018–2019	• Inpatients from wards of Ain Shams University Hospitals, who underwent elective **cardiac surgery** and had **no postoperative complication.**	≥60	180	M: 137 (76.1) F: 43 (23.9)
Wahba, 2013 [66]	Egypt	NR	CS	Convenience	2011–2012	• **Community dwelling** elderly attending the **outpatient** clinics of Ain Shams University Hospitals.	≥60	132	M: 67 (50.7)

									F: 65 (49.3)
Bakr, 2011 [67]	Egypt	100	Prospective	All target population	2009	• Patients that were admitted in different wards in Ain Shams University Hospitals.	≥60	321	NR*
Ismail, 2020 [68]	Lebanon	100	Retrospective	All target population	2009–2015	• Elderly patients who presented to the **emergency department** at a tertiary care center with the chief complaint of "fall".	78.1 (7.2)	253	M: 100 (39.5) F: 153 (60.5)
Musharrafieh, 2011 [69]	Lebanon	NA	Hospital chart review	Convenience	2001–2002	• Geriatric patients presenting to the Emergency Department after a **traumatic** event including those who died on arrival or died in the emergency department.	≥65	62	M: 23 (37.1) F: 39 (62.9)
Zrour, 2020 [70]	Lebanon	NA	Prospective, multi-centric benchmark study	Convenience	2017	• Recruited elderly patients **hospitalized** in surgical floor, medical floor, or intensive care unit from one of the following Lebanese university affiliated hospitals (located in Beirut and its suburbs): Al Makassed Hospital, Sacre Coeur Hospital and Baabda governmental hospital.	75.2 (7.0) for those with absence of delirium 79.0 (7.7) for those with delirium	230	M: 79 (34.4) F: 151 (65.6)
Mahdi, 2017 [71]	Morocco	NR	NR	NR	2014	• Patients diagnosed with **rheumatoid arthritis** at the department of rheumatology, at El Ayachi Hospital, Rabat.	≥61	40	NR*
Bachani, 2011 [72]	Pakistan	NR	National CS	Random	2009**	• Elderly individual in **households** participating to the National Injury Survey of Pakistan.	≥60	1417	M: 795 (56.0) F: 622 (44.0)
Bibi, 2023 [73]	Pakistan	NR	Interventional study	Convenience	2022	• Elderly from two residential areas of Peshawar district, Khyber-Pakhtunkhwa, who have a sedentary lifestyle that is <2 hours activity in 24 hours, with normal to moderate cognitive functioning, able to follow instructions, and able to walk without taking assistance from another person. Baseline data for the random control trail study.	68.1 (50.0), 60–82	140	M: 64 (46.0) F: 76 (54.0)
Fayyaz, 2015 [74]	Pakistan	100	Active surveillance	All target population	2010–2011	• Patients presenting with i**ntentional and unintentional fall-related injuries** in seven major emergency departments (five public and two private hospitals) in six major cities. Data from Pak-NEDS study, a pilot active surveillance carried out in seven major EDs in six major cities of	≥65	78	NR*

						Pakistan including Karachi, Lahore, Islamabad, Rawalpindi, Peshawar, and Quetta.			
Gul, 2021 [75]	Pakistan	NR	CS	Convenience	2014	• Elderly able to walk independently living in household from the Police Colony of Peshawar, Khyber Pakhtunkhwa.	≥60	111	M: 59 (53.0) F: 52 (46.8)
Hashmi, 2013 [76]	Pakistan	NR	CS	Convenience	2012	• Elderly patients with **history of fall** from the emergency units of orthopedic and tertiary care hospitals in Karachi	≥65	100	M: 41 (41.0) F: 59 (59.0)
Naseer, 2022 [77]	Pakistan	NR	CS	Convenience	2020**	• Elderly with from local Pakistani regions in Islamabad and Rawalpindi. Note: **confusing definition of the population** "Individuals with neurological deficiencies or comorbid conditions, a history of falls, fractures or any surgery were excluded from the study". However, all had injuries due to fall.	69.8 (8.5), 60–108	184	M: 103 (56.0) F: 81 (44.0)
Sikander, 2020 [78]	Pakistan	NR	Prospective	Convenience	2019–2020	• Elderly patients with blunt thoracic **trauma** at the Department Thoracic Surgery, Jinnah. Postgraduate Medical Center, Karachi.	70·2 (8·3), 60–88	80	M: 66 (82·5) F: 14 (17·5)
Soomar, 2023 [79]	Pakistan	NR	Cohort study	Purposive	2021–2022	• All elderly patients with a **history of a fall** as a presenting complaint or reason for visiting the Emergency Department.	≥60	318	M: 140 (44.0) F: 178 (56.0)
Tariq, 2013 [80]	Pakistan	NR	CS	NR	2011**	• Elderly living in Karachi recruited from Abbasi Shaheed Hospital, Civil hospital, Jinnah Hospital and Karachi Institute of heart diseases.	≥60	150	M/F (Nb NR)
Halaweh, 2016 a [81]	Palestine	NR	CS	Convenience	2013–2014	• **Community-dwelling** elderly who were independent in indoor ambulation with or without walking aids and living in the West Bank.	68.2 (6.7), 60–91	176	M: 61 (34.7) F: 115 (65.3)
Younis, 2011 [82]	Palestine	100	Retrospective	All target population	2006–2007	• Patients admitted to the emergency departments of 3 hospitals (i.e. Rafidia, Al-Ittihad, and the Specialized Arab hospitals) in Nablus and from the Bethlehem Arab Society for Rehabilitation (BASR) in the southern West Bank, due to **Traumatic Brain Injury**	>65	15	NR*
Braham, 2019 [83]	Tunisia	100	Retrospective	All target population	2016–2017	• Elderly who had an **accidental death** (domestic, work and traffic) and autopsies were performed to determine the unnatural manner and cause of death. Data from police investigation reports and medicolegal	76 (4.3)	239	M: 177 (74.0) F: 62 (26.0)

						autopsy reports collected from the Department of Legal Medicine of Charles Nicolle Hospital in Tunis.			
Abdelkhalik, 2023[84]	Egypt	NR	CS	Purposive	2021–2022	• Elderly individuals in the Ear, Nose & Throat (ENT) and internal medicine **outpatient** clinics at Elsalam hospital, Port Said City. Elderly who were at risk of fall, had a chronic disease and able to walk 10 meters were included.<br>• Elderly with mental disorders, postural hypotension and who had previous hip replacement surgery or a history of lower extremities fracture in the last 12 months were excluded from the research.	68.5 (6.7)	77	M: 49 (63.6)<br>F: 28 (36.4)
Fahim, 2023[85]	Egypt	NR	NR	NR	2023	• Elderly participants who visited the **audio-vestibular clinic** for hearing and/or vestibular evaluation made up the research group. Elderly adults who had neurological disorders, cognitive disorders, or retro labyrinthine lesions were excluded from the research	67.32 (5.4), 60–83	500	M: 318 (63.6)<br>F: 182 (36.4)
Ali Ibrahim, 2023[86]	Egypt	NR	CS	Purposive on all target population	2023	• Elderly **community dwellers** diagnosed with cataract at **outpatient** clinics of the General Ophthalmology Hospital, previously Farouk Hospital, in Alexandria. They had no history of ophthalmic surgeries or glaucoma, no active state of orthopedics, and no neurological disorders such as cerebral vascular stroke and/or Parkinson's disease.<br>Note: All ophthalmologic problems are treated at the facility.	71.8 (7.7), 60–87	100	M: 47 (47.0)<br>F: 53 (53.0)
Salman, 2024[87]	Egypt	NR	CS	Convenience	2022	• Independent elderly patients attending the **outpatient** internal medicine, orthopedics, dental, ophthalmology, ear, nose and throat and Rheumatology clinics at Suez Canal University hospitals.	NR	310	M: 181 (58.4)<br>F: 129 (41.6)
El-Khatib, 2024[88]	Lebanon	NR	CS	Convenience	2022**	• Elderly patients from a wide range of **primary care settings**, including hospitals upon discharge, through home visits, and within **community pharmacies**. Contact was established with both community and hospital pharmacies. Patients were taking five or more medications per day and were able to provide all necessary medical information either directly or with the assistance of a caregiver or family member.	>65	850	M: 419 (49.3)<br>F: 431 (50.7)
Gaber, 2024[89]	Egypt	NR	CS	Convenience	2022	• Elderly residents from the three geriatric homes in Beni-Suef Governorate: El-Khair and El-Baraka Geriatric Home (a government facility) on the East of the Nile; Young Muslim Women Geriatric Home (a government facility) at Mold El Nabi Square; and Red Crescent Geriatric Home (a government facility) on Salah Salem Street.<br>• All residents had either been exposed to the risk of falling or had already experienced a fall.	74.6 (7.8), 60–70	100	M: 61 (61.0)<br>F: 39 (39.0)

LOW-INCOME COUNTRIES									
Abdalla, 2014 [90]	Sudan	99	National CS	Random	2010	• Elderly individual surveyed at national level.<br>Sudan Household Health Survey drawn from the 15 states of Sudan	≥65	3264	NR*

UAE-United Arab Emirates, NA-Not Applicable, NR-Not Reported, CS-Cross-sectional, M-Male, F-Female. *The sex distribution is reported for the whole population and not among the elderly population. ** The year of data collection was not reported but was imputed by adjusting the year of publication based on the median difference between year of publication and the year of data collection (two years).

Table S3: Quality assessment of included studies

Reference	Country	Conflict of Interest/Funding	1	2	3	4	5	6	7	8	9	10
**HIGH-INCOME COUNTRIES**												
Ibrahim, 2021 [1]	Kuwait	None	LR	LR	LR	LR	LR	LR	NA	LR	LR	LR
Al-Balushi, 2012 [2]	Oman	NR	LR	LR	HR	HR	LR	HR	LR	LR	LR	LR
Abdelrahman, 2018 [3]	Qatar	None	LR	LR	LR	LR	LR	HR	NA	LR	LR	LR
Almawlawi, 2011 [4]	Qatar	NR	LR	LR	LR	LR	LR	HR	HR	LR	LR	LR
Alyazeedi, 2019 [5]	Qatar	None	LR	LR	LR	LR	LR	HR	LR	LR	LR	LR
Bener, 2010 [12]	Qatar	None	LR	LR	LR	LR	LR	HR	LR	LR	LR	LR
Bener, 2011 [6]	Qatar	None	LR	LR	LR	LR	LR	HR	NA	LR	LR	LR
Bener, 2012 [7]	Qatar	NR	LR	LR	LR	LR	LR	HR	LR	LR	LR	LR
El-Matbouly, 2013 [8]	Qatar	None	LR	LR	LR	LR	LR	LR	LR	LR	LR	LR
El-Menyar, 2013 [9]	Qatar	None	LR	LR	LR	LR	LR	HR	LR	LR	LR	LR
Hassan, 2023 [10]	Qatar	NR	LR	LR	LR	LR	LR	HR	LR	LR	LR	LR
Mekkodathil, 2020 [11]	Qatar	None	LR	LR	LR	LR	LR	LR	NA	LR	LR	LR
Al Senany, 2015 [13]	Saudi Arabia	None [b]	HR	LR	HR	HR	LR	HR	HR	LR	LR	LR
Alabdullgader, 2021 [14]	Saudi Arabia	None	HR	LR	HR	LR	LR	LR	LR	LR	LR	LR
Alawad, 2020 [15]	Saudi Arabia	None	LR	LR	LR	LR	LR	LR	LR	LR	LR	LR
Aleid, 2023 [32]	Saudi Arabia	None	LR	LR	HR	HR	LR	LR	HR	LR	LR	LR
Alenazi, 2023 [26]	Saudi Arabia	None	LR	LR	HR	HR	LR	LR	HR	LR	LR	LR
Alharbi, 2023 [16]	Saudi Arabia	None	HR	LR	HR	HR	LR	HR	HR	LR	LR	LR
Aljawadi, 2018 [17]	Saudi Arabia	NR	LR	LR	LR	LR	LR	HR	HR	LR	LR	LR
Almegbel, 2017 [18]	Saudi Arabia	None	HR	LR	HR	HR	HR	HR	HR	LR	LR	LR
Al-Qahtani, 2020 [19]	Saudi Arabia	None	LR	LR	LR	HR	LR	HR	LR	LR	LR	LR

Alqarni, 2021[20]	Saudi Arabia	None	HR	LR	LR	LR	LR	HR	LR	LR	LR	LR
Alqurayshah, 2023[33]	Saudi Arabia	None	HR	LR	HR	HR	LR	LR	HR	LR	LR	LR
Alshammari, 2018 [21]	Saudi Arabia	None	LR	LR	HR	HR	LR	HR	LR	LR	LR	LR
Alshehri, 2024[34]	Saudi Arabia	None	LR	LR	HR	HR	LR	LR	HR	LR	LR	LR
Assiri, 2020 [22]	Saudi Arabia	NR	LR	LR	LR	HR	LR	LR	LR	LR	LR	LR
Attar, 2021[23]	Saudi Arabia	None	HR	LR	HR	HR	LR	HR	LR	LR	LR	LR
El-Sobkey, 2011 [24]	Saudi Arabia	NR	HR	LR	HR	HR	LR	LR	NA	LR	LR	LR
Ullah, 2019 [25]	Saudi Arabia	None	HR	LR	LR	LR	LR	LR	LR	LR	LR	LR
Adam, 2008 [27]	UAE	NR	LR	LR	LR	LR	LR	LR	LR	LR	LR	LR
Alao, 2021[28]	UAE	None	LR	LR	LR	LR	LR	LR	LR	LR	LR	LR
Alzaabi, 2022 [29]	UAE	None	LR	LR	HR	HR	LR	LR	HR	LR	LR	LR
Hefny, 2016 [30]	UAE	None	LR	LR	LR	LR	LR	LR	LR	LR	LR	LR
Sharif, 2018 [31]	UAE	None	LR	LR	HR	LR	LR	LR	LR	LR	LR	LR
**UPPER-MIDDLE INCOME COUNTRIES**												
Amin, 2019 [35]	Iraq	NR	HR	LR	HR	HR	LR	LR	NA	LR	LR	LR
Stewart, 2016 [36]	Iraq	None	LR	LR	LR	HR	LR	LR	LR	LR	LR	LR
Muhaidat, 2022 [37]	Jordan	None[c]	LR	LR	HR	HR	LR	HR	HR	LR	LR	LR
**LOWER-MIDDLE INCOME COUNTRIES**												
Adly, 2020 [38]	Egypt	None	HR	LR	HR	HR	LR	HR	LR	LR	LR	LR
Abdelkhalik, 2023[84]	Egypt	NR	HR	LR	HR	HR	LR	LR	HR	LR	LR	LR
Al Tehewy, 2015 [39]	Egypt	NR	HR	LR	LR	LR	LR	LR	LR	LR	LR	LR
Ali Ibrahim, 2023[86]	Egypt	NR	HR	LR	HR	HR	LR	HR	HR	LR	LR	LR
Aly, 2021 [40]	Egypt	None	LR	LR	LR	LR	LR	LR	HR	LR	LR	LR
Amer, 2018 [41]	Egypt	None	HR	LR	HR	HR	LR	HR	HR	LR	LR	LR
Bakr, 2011 [67]	Egypt	NR	HR	LR	LR	LR	LR	HR	LR	LR	LR	LR
El Sayed, 2023 [42]	Egypt	None	HR	LR	HR	HR	LR	HR	LR	LR	LR	LR

El-Gilany, 2011 [43]	Egypt	NR	HR	LR	LR	LR	LR	HR	HR	LR	LR	LR
El-Gilany, 2013 [44]	Egypt	None	HR	LR	HR	HR	LR	HR	NA	LR	LR	LR
El-Kawaly, 2016 [45]	Egypt	NR	HR	LR	HR	HR	LR	HR	HR	LR	LR	LR
El-Rahman, 2014 [46]	Egypt	None	HR	LR	HR	HR	LR	HR	HR	LR	LR	LR
Elsamahy, 2019 [47]	Egypt	NR	HR	LR	HR	HR	LR	HR	HR	LR	LR	LR
Fahim, 2023[85]	Egypt	None	HR	LR	HR	HR	LR	HR	HR	LR	LR	LR
Gaber, 2024[89]	Egypt	NR	HR	LR	HR	HR	LR	HR	NA	LR	LR	LR
Hamed, 2017 [48]	Egypt	NR	LR	LR	LR	LR	LR	LR	HR	LR	LR	LR
Ismail, 2018 [49]	Egypt	NR	LR	LR	HR	HR	LR	HR	LR	LR	LR	LR
Kamel, 2013 [50]	Egypt	None	HR	LR	HR	HR	HR	LR	HR	LR	LR	LR
Khater, 2012 [51]	Egypt	None	HR	LR	HR	LR	LR	LR	HR	LR	LR	LR
Mabrouk, 2003 [52]	Egypt	NR	HR	LR	HR	HR	LR	HR	LR	LR	LR	LR
Mahmoued, 2014 [53]	Egypt	NR	LR	LR	HR	HR	LR	LR	LR	LR	LR	LR
Makhlouf, 2000 [54]	Egypt	NR	LR	LR	HR	HR	LR	LR	HR	LR	LR	LR
Mohamed, 2019 [55]	Egypt	NR	LR	LR	HR	HR	LR	LR	HR	LR	LR	LR
Mohammed, 2018 [56]	Egypt	None	LR	LR	LR	LR	LR	HR	HR	LR	LR	LR
Mohammed, 2019 [57]	Egypt	None	HR	LR	HR	LR	LR	LR	LR	LR	LR	LR
Mohsen, 2019 [59]	Egypt	None	LR	LR	LR	HR	LR	HR	NA	LR	LR	LR
Mohammed, 2021 [58]	Egypt	NR	HR	LR	HR	HR	LR	LR	HR	LR	LR	LR
Saleh, 2018 [61]	Egypt	NR	LR	LR	LR	HR	LR	HR	HR	LR	LR	LR
Sakr, 2006 [60]	Egypt	NR	HR	LR	LR	LR	LR	HR	NA	LR	LR	LR
Saudi, 2021[62]	Egypt	None	HR	LR	HR	HR	LR	HR	HR	LR	LR	LR
Sayed, 2015 [63]	Egypt	NR	HR	LR	HR	HR	LR	HR	HR	LR	LR	LR
Salman, 2024[87]	Egypt	NR	HR	LR	HR	HR	LR	HR	HR	LR	LR	LR
Sharaf, 2008 [64]	Egypt	NR	LR	LR	LR	LR	LR	HR	HR	LR	LR	LR
Tawfik, 2021[65]	Egypt	None	HR	LR	LR	LR	LR	HR	LR	LR	LR	LR

Wahba, 2013 [66]	Egypt	None	HR	LR	HR	HR	LR	HR	HR	LR	LR	LR
El-Khatib, 2024[88]	Lebanon	None	LR	LR	HR	HR	LR	HR	HR	LR	LR	LR
Ismail, 2020 [68]	Lebanon	None	LR	LR	LR	LR	LR	LR	LR	LR	LR	LR
Musharrafieh, 2011 [69]	Lebanon	NR	HR	LR	HR	HR	LR	LR	LR	LR	LR	LR
Zrour, 2020 [70]	Lebanon	None	LR	LR	HR	HR	LR	LR	LR	LR	LR	LR
Mahdi, 2017 [71]	Morocco	None	HR	LR	HR	HR	LR	HR	HR	LR	LR	LR
Bachani, 2011 [72]	Pakistan	NR	LR	LR	LR	HR	LR	HR	HR	LR	LR	LR
Bibi, 2023 [73]	Pakistan	None	HR	LR	HR	HR	LR	HR	LR	LR	LR	LR
Fayyaz, 2015 [74]	Pakistan	None	LR	LR	LR	LR	LR	LR	LR	LR	LR	LR
Gul, 2021 [75]	Pakistan	None	HR	LR	HR	HR	LR	HR	HR	LR	LR	LR
Hashmi, 2013 [76]	Pakistan	NR	HR	LR	HR	HR	LR	LR	NA	LR	LR	LR
Naseer, 2022 [77]	Pakistan	None	HR	HR	HR	HR	LR	HR	HR	LR	LR	LR
Sikander, 2020 [78]	Pakistan	None	HR	LR	HR	HR	LR	HR	LR	LR	LR	LR
Soomar, 2023 [79]	Pakistan	None	HR	LR	HR	HR	LR	LR	LR	LR	LR	LR
Tariq, 2013 [80]	Pakistan	NR	LR	LR	HR	HR	LR	HR	HR	LR	LR	LR
Halaweh, 2016 a [81]	Palestine	NR	HR	LR	HR	HR	LR	LR	LR	LR	LR	LR
Younis, 2011 [82]	Palestine	None	HR	LR	LR	LR	LR	HR	LR	LR	LR	LR
Braham, 2019 [83]	Tunisia	None	LR	LR	LR	LR	LR	HR	LR	LR	LR	LR
**LOW-INCOME COUNTRIES**												
Abdalla, 2014 [90]	Sudan	None	LR	LR	LR	LR	LR	LR	LR	LR	LR	LR

Notes:
Risk of Bias in Prevalence studies tool[91]

1. Was the study's target population a close representation of the national population in relation to relevant variables?
2. Was the sampling frame a true or close representation of the target population?
3. Was some form of random selection used to select the sample, OR was a census undertaken?
4. Was the likelihood of nonresponse bias minimal?
5. Were data collected directly from the subjects (as opposed to a proxy)?
6. Was an acceptable case definition used in the study?

7a. Was the study instrument that measured the parameter of interest (Prevalence) shown to have validity and reliability?
7b. Was the study instrument that measured the parameter of interest (Risk of fall) shown to have validity and reliability?
7c. Was the study instrument that measured the parameter of interest (Fear of fall) shown to have validity and reliability?
8. Was the same mode of data collection used for all subjects?
9. Was the length of the shortest prevalence period for the parameter of interest appropriate?
10. Were the numerator(s) and denominator(s) for the parameter of interest appropriate?

LR= Low Risk of Bias; HR= High Risk of Bias; NA= Not Applicable.

[a]The authors would like to thank the Deanship of Scientific Research at Umm Al-Qura University for supporting this work by Grant Code: 22UQU4280521DSR01.
[b]This project was funded by the Deanship of Scientific Research (DSR), King Abdulaziz University, Jeddah, Saudi Arabia under grant No. (142-004-D1434). The authors, therefore, acknowledge with thanks DSR technical and financial support.
[c]The study was funded by the Deanship of Academic Research at The University of Jordan (grant number Deanship of Academic Research, The University of Jordan 1517).
[d]This study was funded by the University Kebangsaan Malaysia Medical Centre Fundamental Research Grant, Code Number FF-017-2012 without which the study would not have been possible.

Figure S1: Summary of the quality assessment of the included primary studies

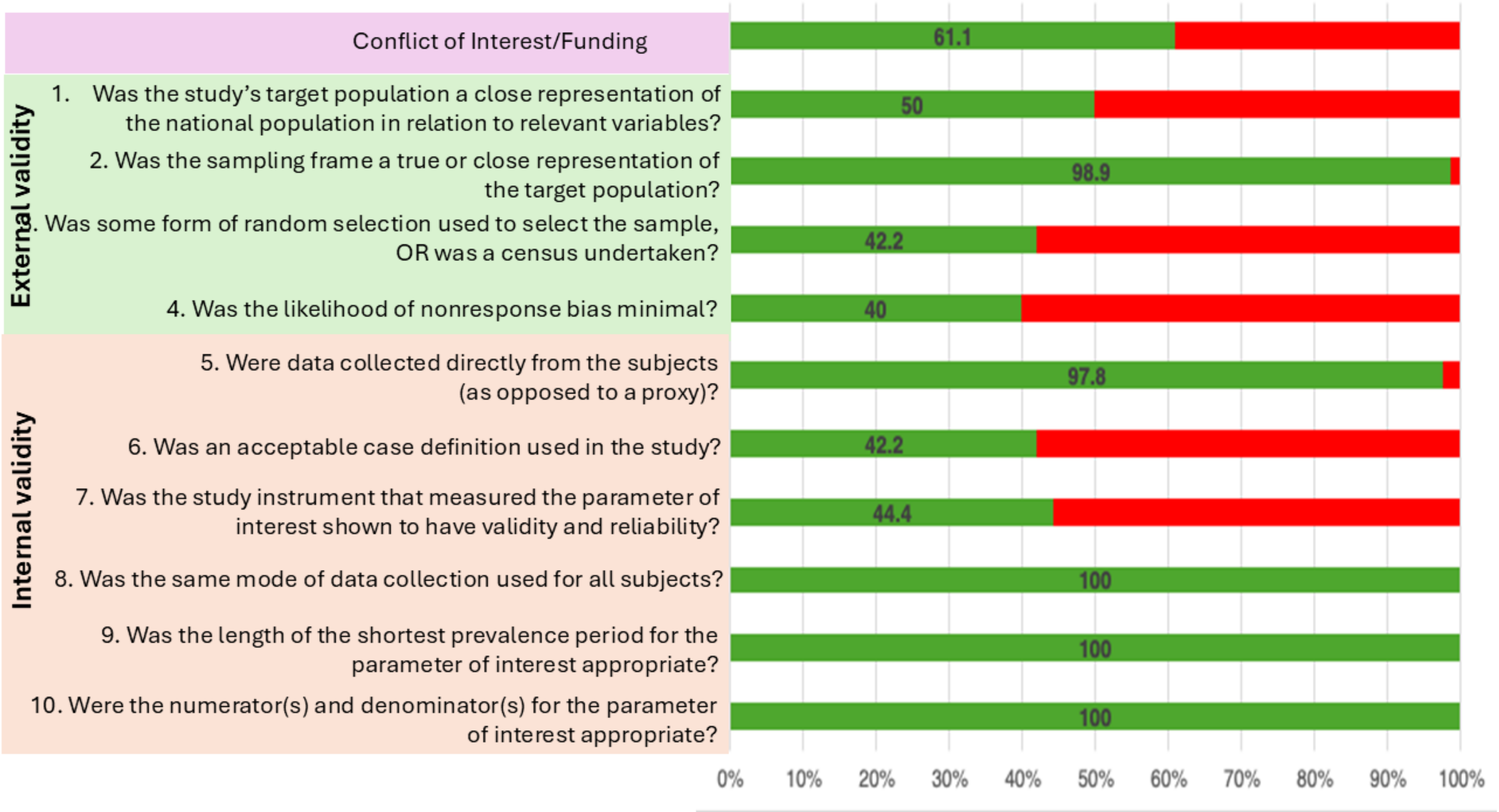

Green bar: low risk of bias, red bar: high risk of bias

Box S3: Study selection for the meta-analysis

- Naseer et al., 2022[77] had a confusing definition of the population, therefore was excluded from the meta-analysis of prevalence of fallers among elderly but included in the meta-analysis of consequences.
- Adam et al., 2008[27]was excluded from the meta-analysis because the population studied was a subgroup of a population studied in Hefny et al., 2016 [30].
- Assiri et al., 2020[22] was excluded from the age-specific subgroup analysis because the reported age groups did not match the 60-69 and ≥70 format.
- Three studies (Attar et al., 2021[23], Soomar et al., 2023[79], and Fayyaz et al., 2015[74]) were conducted among elderly fallers and were excluded from the meta-analysis. Two studies reported prevalence of previous falls among the current fallers ((Attar et al., 2021[23], Soomar et al., 2023[79]), and the other reported the proportion of the elderly who experienced unintentional falls out of all types of falls (Fayyaz et al., 2015[74]).
- Khater et al., 2012 [51] provided the prevalence of fallers among the elderly living in nursing homes during the past ("previous falls") and after one year of follow-up. To minimize recall bias, the one-year follow-up measure was the one utilized in the meta-analysis estimating the pooled prevalence of fallers among the elderly within a one-year time frame.
- Alabdullgader et al.,2021[14] reported the prevalence of fallers among elderly outpatients in the past 3 months and in the past year. The measure for the past 3 months was excluded from the meta-analysis.
- Alshammari et al., 2018 [21] was excluded from the meta-analysis as it was the only study reporting the prevalence of fallers among community-dwelling elderly in the past 3 months
- Halaweh et al., 2016a[81] was excluded from the meta-analysis as it was the only study reporting the prevalence of fallers among community-dwelling elderly in the past 6 months
- Sharif et al., 2018[31] was excluded from the meta-analysis as it was the only study reporting the prevalence of fallers among community-dwelling elderly in the past 2 years
- Mohammed et al., 2021[58] reported on a mixed population including 'geriatric' homes' residents and community dwelling outpatients. This study was excluded because of the mixed population and because the time frame was 6 months.
- Muhaidat et al., 2022[37] recruited community dwelling elderly from mixed setting including community setting and waiting rooms of outpatient's clinics. This population was considered as the general population
- Bachani et al., 2011[72] reported 0 fallers among 795 male elderly. Musharrafieh et al., 2011[69] reported 0 fallers from more than a height of 15ft, among 62 trauma patients. To consider these measures in the subgroup meta-analysis, 1 was added as the number of cases to allow meta-analysis computation.
- When prevalence was 0%, 1 was added to the number of events to allow the inclusion of the studies (Sharif et al., 2018[31] and Hefny et al., 2016 [30]) in the meta-analysis of consequences.

Table S4: Prevalence of fall in the older general population, Time frame: 1 year.

Category	Number of studies	Sample Size	Number of Fallers	Prevalence Range (%)	Pooled Estimate [95% CI]	Heterogeneity measures		Q between subgroup tests (p-value)
						Q p-value	I^2(%)	
**Overall (5 countries)**	15	9880	1950	1.6-63.0	28.5 [18.2-41.6]	<0.0001	98.9	NA
**World Bank Income Levels**								
Low Income	1	3264	51	0-1.6	1.6 [1.2-2.0]	NA	NA	<0.0001
High Income	7	4324	1032	9.0-52.3	28.1 [16.2-44.1]	<0.0001	99	
Lower-Middle Income	5	2172	825	32.2-63.0	40.8 [32.3-49.8]	<0.0001	89.5	
Upper-Middle Income	2	120	42	33.3-35.8	35.0 [27.0-43.9]	<0.0001	0	
**Sex**								
Male	5	2511	562	9.0-47.2	28.8 [16.8-44.8]	<0.0001	98.8	0.4545
Female	5	2871	850	17.5-52.3	36.2 [24.7-49.6]	<0.0001	98.4	
**Age (years)**								
60-69	3	2321	292	10.8-19.7	13.8 [9.7-19.3]	<0.0001	92.1	0.0013
≥70	4	1308	483	16.7-66.7	39.1 [23.4-57.5]	<0.0001	94.2	
**Time period**								
2006-2017	9	9292	1758	1.6-63.0	26.5 [12.8-46.9]	<0.0001	99.3	0.6012
2017-2022	6	588	192	12.5-52.0	32.1 [22.8-43.0]	<0.0001	79.7	

Table S5: Prevalence of a history of fall among the older general population in MENA.

Category	Number of studies	Sample Size	Number of Fallers	Prevalence Range (%)	Pooled Estimate [95% CI]	Heterogeneity measures		Q between subgroup tests (p-value)
						Q p-value	I^2(%)	
**Overall (5 countries)**	13	3462	884	0.1-76.9	22.4 [8.2-48.4]	<0.0001	96.8	NA
**World Bank Income Levels**								
Lower-Middle Income	12	3234	869	0.1-76.9	24.5 [8.5-53.2]	<0.0001	96.1	0.0273
Upper-Middle Income	1	228	15	0-6.6	6.6 [3.7-10.6]	NA	NA	
**Sex**								
Male	3	965	66	0.1-41.5	7.1 [0.3-68.9]	<0.0001	94.8	0.6411
Female	3	828	136	0.5-76.9	18.8 [0.9-85.2]	<0.0001	98.3	
**Age (years)**								
60-69	4	574	229	10.3-54.4	33.5 [17.9-53.8]	<0.0001	92.1	0.3241
≥70	4	279	161	24.6-71.9	48.8 [26.9-71.2]	<0.0001	92.4	
**Time period**								
2003-2015	9	2694	574	0.1-76.9	16.2 [3.5-51.1]	<0.0001	97.8	0.1470
2016-2022	4	768	310	32.8-43.4	40.4 [36.9-43.9]	<0.0001	0	

MENA: Middle East and North Africa

Figure S2: Prevalence of fallers in the past year among the older population living in community, by country

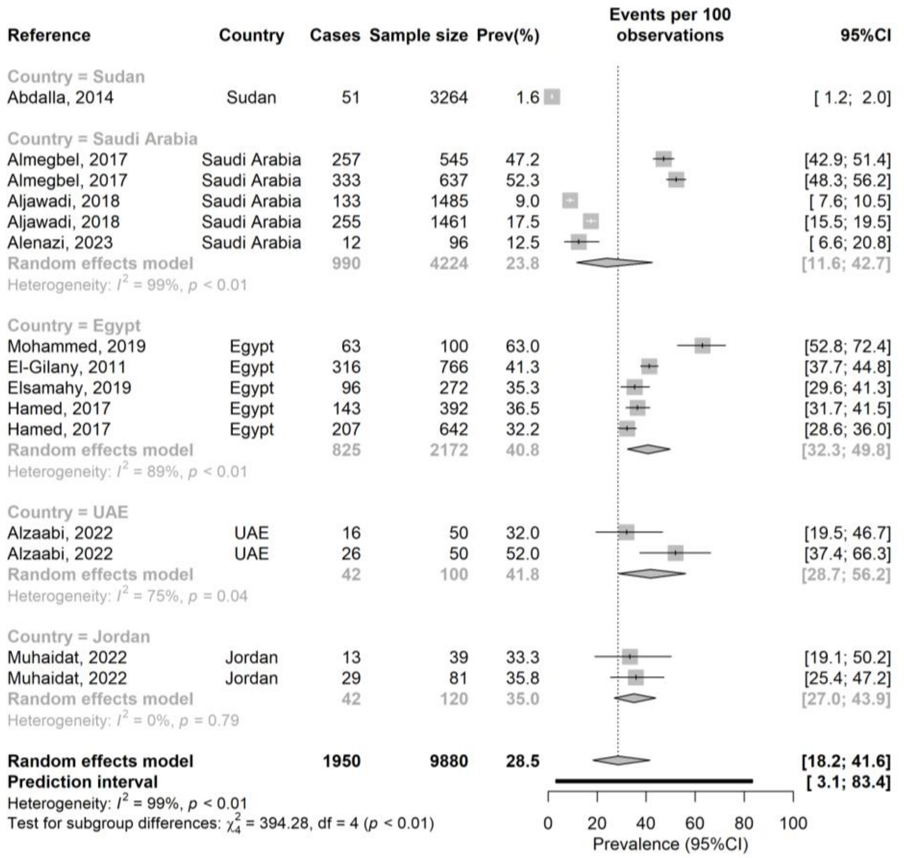

Figure S3: Prevalence of history of fall among the older population living in community, by country

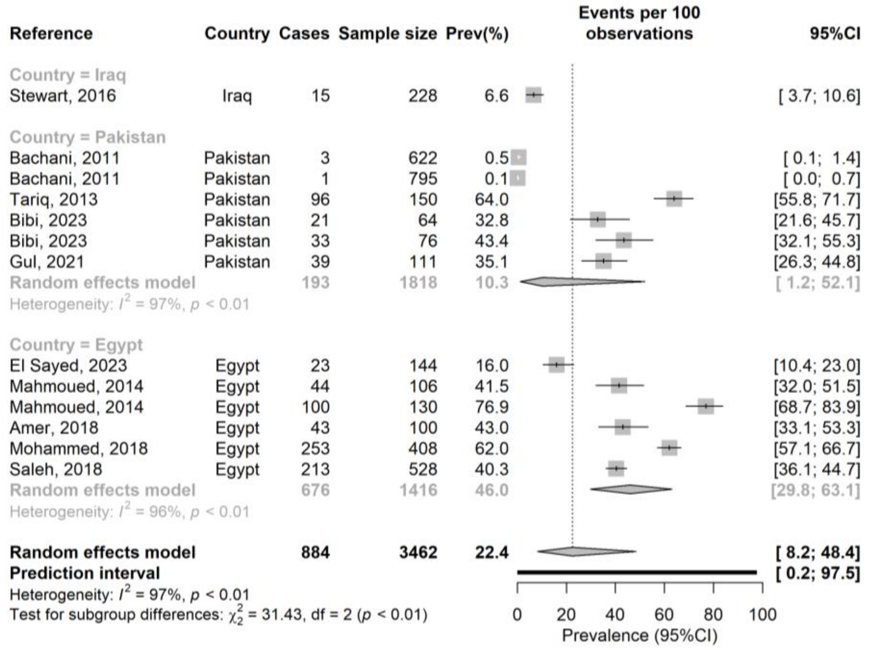

Table S6: Weighted average pooled proportion of fall frequency among the older people in MENA.

Frequency	Number of studies	Sample Size	Number of Fallers	Prevalence Range (%)	Pooled Estimate [95% CI]	Heterogeneity measures		Q between subgroup tests (p-value)
						Q p-value	I^2(%)	
Time frame: past one year								
1	17	2098	1130	19.0-79.0	54.0 [46.0-61]	<0.01	84.4	0.68
≥2	17	2078	1057	21.0-100.0	59.0 [40.0-76.0]	<0.01	84.3	
Time frame: history of fall								
1	5	621	280	31.9-93.5	52.7 [28.1-76.0]	<0.01	91.2	0.7756
≥2	5	621	341	6.5-68.1	47.3 [24.0-71.9]	<0.01	91.2	

Figure S4: Prevalence of fallers among older adults living in residential homes in Egypt.

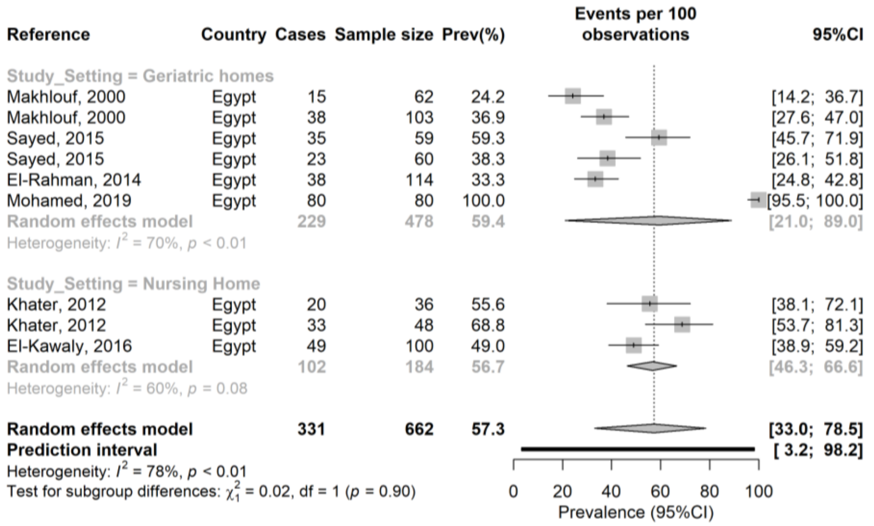

Time frame: past 1 year

Table S7: Prevalence of falls among the older people in residential homes in Egypt.

Category	Number of studies **	Sample Size	Number of Fallers	Prevalence Range (%)	Pooled Estimate [95% CI]	Heterogeneity measures		Q between subgroup tests (p-value)
						Q p-value	I^2(%)	
**Overall***	9	662	331	24.2-100.0	57.3 [33.0-78.5]	<0.0001	78.1	NA
**Sex***								
Male	3	157	70	24.2-59.3	45.4 [27.4-64.7]	<0.0001	87.8	0.8815
Female	3	211	94	36.9-68.8	47.4 [31.4-63.9]	<0.0001	85.5	
**Age (years)***								
60-69	2	128	40	12.8-42.0	25.6 [10.0-51.6]	<0.0001	90.5	0.1158
≥70	2	156	71	39.8-63.2	49.6 [33.6-65.7]	<0.0001	83.6	
**Time Period***								
1998-1999	2	165	53	24.2-36.9	31.6 [23.3-41.2]	<0.0001	64.6	0.0005
2009-2010	2	84	53	55.6-68.8	63.1 [52.3-72.7]	<0.0001	34.5	
2013-2015	4	333	145	33.3-59.3	44.4 [34.9-54.4]	<0.0001	75.8	
2016-2018	1	80	80	0-100	100 [95.5-100.0]	NA	NA	
**Time Frame**								
Past 6 months	1	120	43	0-35.8	35.8 [27.3-45.1]	NA	NA	<0.01
Past 1 year	7	578	278	24.2-100	57.0 [25.0-84.0]	<0.0001	71.2	
Past (“history since age of 60”)	1	208	116	0-55.8	55.8 [48.7-62.6]	NA	NA	
Past (Time NR)	2	184	148	59.5-98.0	89.3 [39.3-99.1]	<0.0001	95.4	
1 year follow-up	2	84	53	55.6-68.8	63.1 [52.3-72.7]	<0.0001	34.5	

* The time frame of the included studies is 1 Year ** All included studies were from one country (Egypt)
NA-Not Applicable NR-Not Reported MENA-Middle East and North Africa

Figure S5: Prevalence of fallers among older traumatic patients, by country.

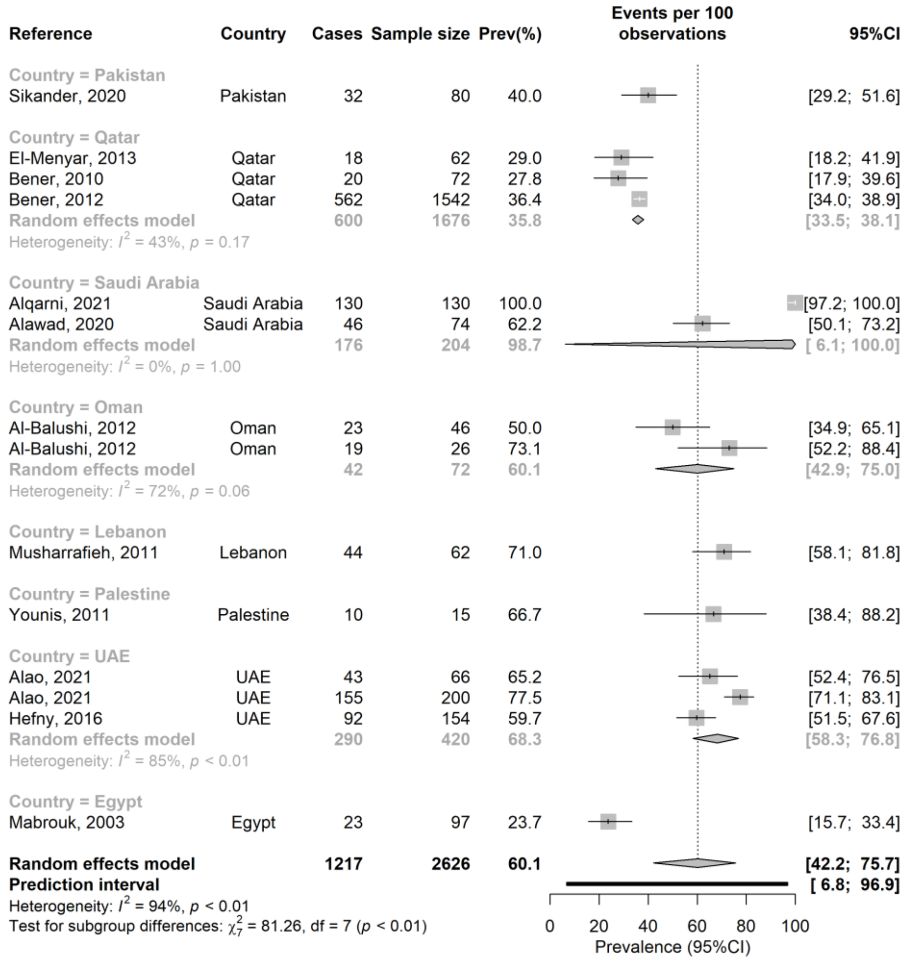

Time frame: current falls

Figure S6: Prevalence of fallers among older outpatients, by country.

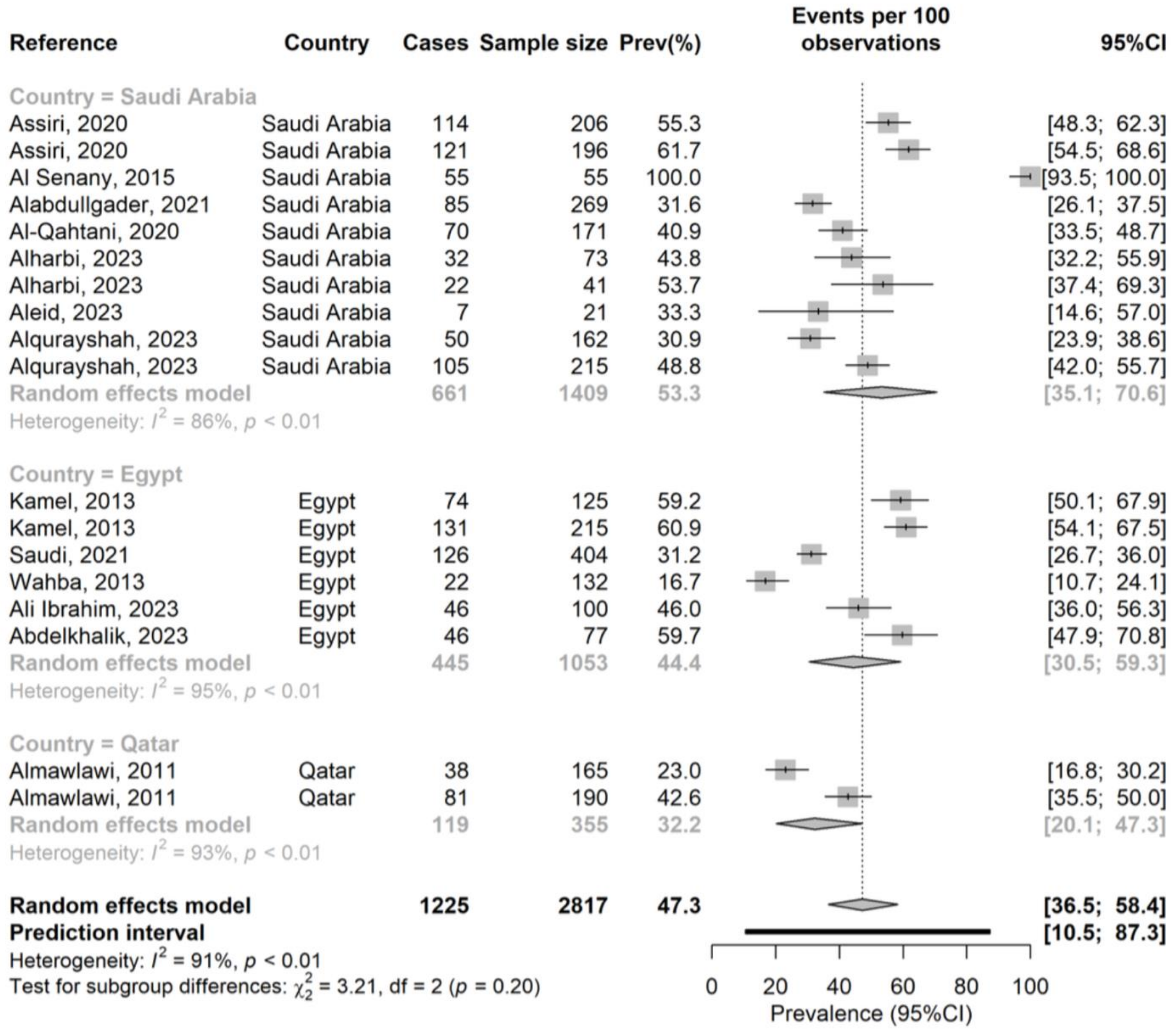

Time frame: past 1 year

Figure S7: Prevalence of falls among the older clinical population in MENA, by type of fall.

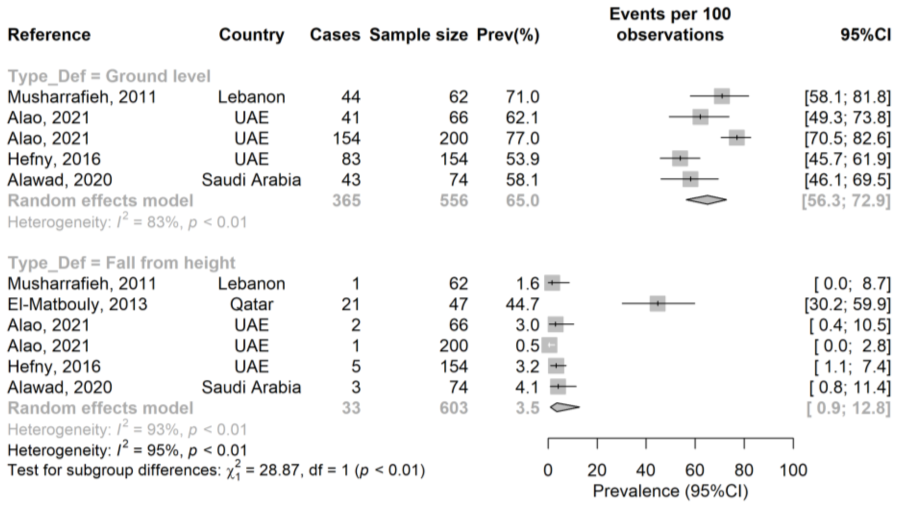

Time frame: past 1 year and current falls

Table S8: Results of meta-regressions aimed at identifying associations and sources of between-study heterogeneity in the prevalence of fallers among the older population in MENA.

	Studies (n)	Samples (n)	Univariable analyses			Unaccounted variability I2	Multivariable analysis	
			OR (95% CI)	p value	LR test p value		Adjusted OR (95% CI)	p value
**Population type**								
Community dwelling	28	13342	1·00 (ref)	..	<0·0001	98.80%	1·00 (ref)	..
Outpatients	18	2817	2.55 (0.02–9.54)	**0.0241**	..	..	1.65 (0.73–3.74)	0.2228
Residential homes	9	662	3.54 (1.25–10.05)	**0.0172**	..	..	2.04(0.72–5.75)	0.1746
Trauma patients	14	2626	4.14 (1.70–10.07)	**0.0017**	..	..	2.87 (1.14–7.24)	**0.0246**
**Sex**								
Women	17	4793	1·00 (ref)	..	<0·0001	98.99%	..	..
Men	18	4581	0.63 (0.23–1.68)	0.3586	..	..	..	..
Men & women	34	10073	1.20 (0.50–2.85)	0.6744	..	..	..	..
**World Bank income levels**								
High income	29	8460	1·00 (ref)	..	<0·0001	98.87%	1·00 (ref)	..
Upper-middle income	3	348	0.280 (0.05–1.47)	0.1326	..	..	0.46 (0.09–2.24)	0.3387
Lower-middle income	36	7375	0.723 (0.36–1.42)	0.3516	..	..	0.72 (0.35–1.48)	0.3787
Low income	1	3264	0.01 (0.00–0.25)	**0.0034**	..	..	0.05 (0.00–0.73)	**0.0275**
**Diagnosis**								
Clinical	21	6745	1·00 (ref)	..	<0·0001	98.98%	..	..
Self-reported	48	12702	0.510 (0.23–1.08)	0.0822	..	..		..
**Validated instruments**							..	
Validated	30	9182	1·00 (ref)		<0·0001	99.00%	..	..
Non-validated	39	10265	0.61 (0.30–1.24)	0.1737	..	..	..	..
**Age definition**								
≥60	53	14417	1·00 (ref)	..	<0·0001	99.06%	..	..
65	16	5030	1.40 (0.60–3.26)	0.4285	..	..	..	..
**Sample size**								
≥200	21	15096	1·00 (ref)	..	<0·0001	98.91%	1·00 (ref)	..
<200	48	4351	0.32 (0.15–0.66)	**0.0020**	..	..	0.64 (0.30–1.35)	0.2455
**Response rate**								
≥80%	18	7512	1·00 (ref)	..	<0·0001	99.03%	..	
<80% or unclear	51	11935	1.06 (0.47–2.40)	0.8701	..	..	..	..
**Time frame**								
One year	42	13359	1·00 (ref)	..	<0·0001	98.87%	..	
Current status	14	2626	2.03 (0.85–4.85)	0.1078	..	..	..	..
History	13	3462	0.42 (0.17–1.03)	0.0585	..	..	..	
**Sampling methodology**								
All target population or random	22	12802	1·00 (ref)	..	<0·0001	98.95%	1·00 (ref)	..
Non-probability or unclear	47	6645	2.43 (1.17–5.05)	**0.0171**			2.023 (0.98–4.16)	0.0558
**Year of data collection**	69	19447	1.03 (0.97–1.09)	0.2733	<0·0001	99.03%	..	..

Table S9: Additional reported proportions of older fallers by health consequence after a fall in the Middle East and North Africa

**Consequences**	**Number of studies**	**Sample Size of fallers**	**Nb of Fallers reporting that consequences**	**Prevalence Range (%)**
Fracture of limb [56]	1	253	32	0-12.6
Fracture of femur and hip [56]	1	253	45	0-17.8
Stress fracture [18]	1	590	47	0-8.0
Burn Injury [6]	1	134	1	0-0.7
Non-traumatic Injury [35]	1	150	55	0-36.7
Haemorrhage [48]	1	350	38	0-10.9
Intracranial haemorrhage [31]	1	188	1	0-0.5
Fear of future falls [58]	1	244	243	0-99.6
Reduced activity [48]	1	350	91	0-26.0
Loss of consciousness [18]	1	590	35	0-5.9
“Ability to independently return to previous position” [18]	1	590	295	0-50.0
Affect light activities such as running, lifting light objects and participating in all kinds of sports [89]	1	100	29	0-29.0
Affect meeting your own needs (groceries or shopping) [89]	1	100	74	0-74.0
Affect showering and getting dressed alone [89]	1	100	67	0-67.0
Affect the daily personal hygiene practice [89]	1	100	70	0-70.0
Affect your ascent of all stairs [89]	1	100	88	0-88.0
Affect your daily life and homework practices [89]	1	100	85	0-88.0
Affect your going to work [89]	1	100	63	0-63.0
Became a person unable to cope with problems and developments well [89]	1	100	12	0-12.0
Become hostile to others and yourself? [89]	1	100	82	0-82.0
Blame others for their lack of caring [89]	1	100	69	0-69.0

Did you feel tired? [89]	1	100	50	0-50.0
Did you feel worn out? [89]	1	100	85	0-0.85.0
Disability or distortion [89]	1	100	72	0-72.0
Have you been a happy person? [89]	1	100	30	0-30.0
Sleep [89]	1	100	78	0-78.0
Stress [89]	1	100	2	0-2.0
Stress fracture [18]	1	590	47	0-47.0
The impact of the fall on bending or kneeling [89]	1	100	80	0-80.0

Table S10: Weighted average pooled proportion of self-reported perceived reason of falls among the older population in MENA.

Causes	Number of studies	Sample Size	Number of Fallers	Prevalence Range (%)	Pooled Estimate [95% CI]	Heterogeneity measures	
						Q p-value	I^2(%)
Dizziness, Balance, Drop attack, Vertigo	11	1386	468	5.0-82.0	29.6 [17.8-45.0]	<0.01	93.1
Slipping, Tripping	8	1075	326	9.0-54.0	34.3 [22.0-49.2]	<0.01	95.0
Environment	7	660	203	6.0-93.0	32.0 [12.0-62.0]	<0.01	94.3
Medical condition	6	519	263	7.0-87.0	37.2 [13.0-70.0]	<0.01	96.9
Other, unknown cause, no cause	4	566	94	9.4-19.3	16.6 [13.7-19.9]	0.26	24.6
Vision problem	5	475	193	11.7- 89.1	51.2 [21.6-79.9]	<0.01	97.0
Drowsiness Medications	3	418	135	10.1-63.0	34.0 [12.0-65.0]	<0.01	97.3
Environment and Slipping Tripping	2	230	134	57.0-59.2	58.2 [51.7-64.4]	0.73	0
Hearing Problem	2	141	18	12.2-13.9	12.7 [8.1-19.3]	0.78	0
Surgical	2	141	17	8.1-20.9	12.7 [6.3-23.7]	0.04	76.8
Syncope fainting	2	205	8	1.8-4.6	3.9 [1.9-7.6]	0.37	0
Unintentionally moving out of bed	2	280	31	7.6-14.0	10.8 [7.1-16.2]	0.10	63.5
Knee gave away	1	150	11	0-7.0	7.3 [4.1-12.7]	NA	NA
Lack of awareness	1	100	51	0-51.0	51.0 [41.2-60.6]	NA	NA
Muscle weakness	1	100	79	0-79.0	79.0 [69.9-85.8]	NA	NA
Insensitivity and weakness in the feet	1	100	90	0-90.0	90.0 [92.3-99.5]	NA	NA
Aging	1	100	98	0-98.0	98.0 [92.3-99.5]	NA	NA

Figure S8: doi plot assessing publication bias

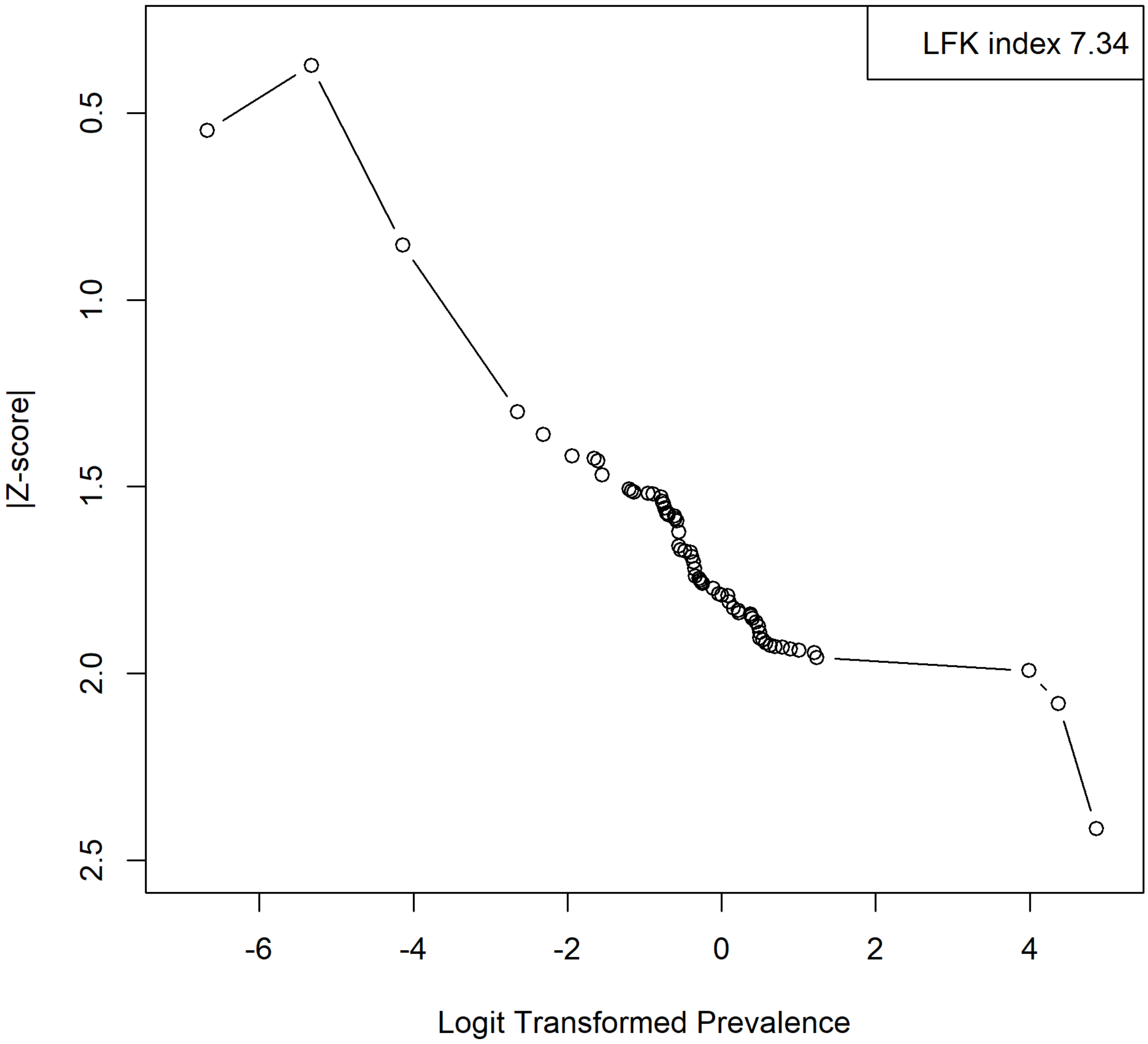

Note: doi plot shows normal-quantile (Z-score) against logit transformation of prevalence (X-axis), and LFK index to assess publication bias by visualizing asymmetry and quantifying potential asymmetry of study effects[92]. LFK index=7.34 suggests a tendency for studies for reporting higher prevalences of falls to be more frequently published.

Reference list:

1 Ibrahim IK, AlAsoomi F. Hospitalization of unintentional fall injuries in Kuwait: a national database study. BMC public health. 2021;21:1364.

2 Al-Balushi H, Al-Kalbani A, Al-Khwaldi T, Al-Suqri S, Al-Maniri A, Alazri M, et al. Injuries presented at a primary care setting in oman. Oman medical journal. 2012;27:486-90.

3 Abdelrahman H, Almadani A, El-Menyar A, Shunni A, Consunji R, Al-Thani H. Home-related falls: An underestimated mechanism of injury. Journal of Family and Community Medicine. 2018;25:48-51.

4 Almawlawi E, Al Ansari A, Ahmed A. Prevalence and risk factors for falls among the elderly in primary healthcare centers (PHC) in Qatar. Qatar medical journal. 2011;2011:7.

5 Alyazeedi A, Fouad Algendy A, Sharabash M, Karawia A. Prevalence, Determinants And Associated Risk Of Potentially Inappropriate Prescribing For Older Adults In Qatar: A National Retrospective Study. Clinical interventions in aging. 2019;14:1889-99.

6 Bener A, Abdul Rahman YS, Abdel Aleem EY, Khalid MK. Trends and Characteristics of Head and Neck Injury from Falls: A hospital based study, Qatar. Sultan Qaboos University medical journal. 2011;11:244-51.

7 Bener A, Abdul Rahman YS, Abdel Aleem EY, Khalid MK. Trends and characteristics of injuries in the State of Qatar: hospital-based study. International journal of injury control and safety promotion. 2012;19:368-72.

8 El-Matbouly M, El-Menyar A, Al-Thani H, Tuma M, El-Hennawy H, AbdulRahman H, et al. Traumatic brain injury in Qatar: age matters--insights from a 4-year observational study. TheScientificWorldJournal. 2013;2013:354920.

9 El-Menyar A, Latifi R, AbdulRahman H, Zarour A, Tuma M, Parchani A, et al. Age and traumatic chest injury: a 3-year observational study. European Journal of Trauma and Emergency Surgery. 2013;39:397-403.

10 Hassan DA, Syed M, Shraim M. Prevalence and factors associated with falls in older adults in a middle eastern population: A cross-sectional study. Population Medicine. 2023;5.

11 Mekkodathil A, El-Menyar A, Kanbar A, Hakim S, Ahmed K, Siddiqui T, et al. Epidemiological and clinical characteristics of fall-related injuries: a retrospective study. BMC public health. 2020;20:1186.

12 Bener A, Omar AO, Ahmad AE, Al-Mulla FH, Abdul Rahman YS. The pattern of traumatic brain injuries: a country undergoing rapid development. Brain injury. 2010;24:74-80.

13 Al Senany S, Al Saif A. Assessment of physical health status and quality of life among Saudi older adults. Journal of Physical Therapy Science. 2015;27:1691-5.

14 Alabdullgader A, Rabbani U. Prevalence and Risk Factors of Falls Among the Elderly in Unaizah City, Saudi Arabia. Sultan Qaboos University medical journal. 2021;21:e86-e93.

15 Alawad MO, Alenezi N, Alrashedan BS, Alsabieh M, Alnasser A, Abdulkader RS, et al. Traumatic spinal injuries in Saudi Arabia: a retrospective single-centre medical record review. BMJ open. 2020;10:e039768.

16 Alharbi AA. Comparison of three fall risk assessment tools in community-dwelling Saudi elderlies: A prospective study. 2023.

17 Aljawadi MH, Khoja AT, Alhammad AM, AlOtaibi AD, Al-Shammari SA, Khoja TA. The prevalence of benzodiazepines utilization and its association with falls among Saudi older adults; results from the Saudi national survey for elderly Health (SNSEH). Saudi pharmaceutical journal : SPJ : the official publication of the Saudi Pharmaceutical Society. 2018;26:1112-9.

18 Almegbel FY, Alotaibi IM, Alhusain FA, Masuadi EM, Al Sulami SL, Aloushan AF, et al. Period prevalence, risk factors and consequent injuries of falling among the Saudi elderly living in Riyadh, Saudi Arabia: a cross-sectional study. BMJ open. 2018;8:e019063.

19 Al-Qahtani AM. Health Status and Functional Abilities of Elderly Males Visiting Primary Health-care Centers in Khamis Mushait, Saudi Arabia. Clinical interventions in aging. 2020;15:2129-43.

20 Alqarni MS, Bukhari ZM, Alsinnari Y, Attar M, Alzahrani A, Abukhodair AW, et al. 30-day mortality rate following hip fractures in elderly patients admitted to a tertiary care center. Australasian Medical Journal. 2021;14:118-22.

21 Alshammari SA, Alhassan AM, Aldawsari MA, Bazuhair FO, Alotaibi FK, Aldakhil AA, et al. Falls among elderly and its relation with their health problems and surrounding environmental factors in Riyadh. Journal of family & community medicine. 2018;25:29-34.

22 Assiri EH, Assiri MH, Alsaleem SA, Assiri AH, Assiri AH, Ahmed RA, et al. Epidemiology of falls among elderly people attending primary healthcare centers in Abha City, Saudi Arabia. World Family Medicine. 2020;18:47-59.

23 Attar M, Alsinnari YM, Alqarni MS, Bukhari ZM, Alzahrani A, Abukhodair AW, et al. Common Types of Falls in the Elderly Population, Their Associated Risk Factors and Prevention in a Tertiary Care Center. Cureus. 2021;13:e14863.

24 El-Sobkey SB. Balance performance of community-dwelling older people. Saudi medical journal. 2011;32:283-7.

25 Ullah S, Al-Atwi MK, Qureshi AZ, Tantawy SS, Ilyas A, Wunderlich CA. Falls in individuals with stroke during inpatient rehabilitation at a tertiary care hospital in Saudi Arabia. Neurosciences (Riyadh, Saudi Arabia). 2019;24:130-6.

26 Alenazi AM. Number of medications and polypharmacy are associated with risk of fall in Saudi community-dwelling adults. Saudi pharmaceutical journal. 2023;31:185-90.

27 Adam SH, Eid HO, Barss P, Lunsjo K, Grivna M, Torab FC, et al. Epidemiology of geriatric trauma in United Arab Emirates. Archives of gerontology and geriatrics. 2008;47:377-82.

28 Alao DO, Cevik AA, Grivna M, Eid HO, Abu-Zidan FM. Epidemiological changes of geriatric trauma in the United Arab Emirates2021 2021-6-4.

29 Alzaabi HS, Walton LM, Arumugam A. Association between demographic characteristics, lower limb range of motion, functional performance, ability to dual task, quality of life and risk of falls in older adults of the United Arab Emirates-A cross-sectional study. Heliyon. 2022;8:e08869.

30 Hefny AF, Abbas AK, Abu-Zidan FM. Geriatric fall-related injuries. African health sciences. 2016;16:554-9.

31 Sharif SI, Al-Harbi AB, Al-Shihabi AM, Al-Daour DS, Sharif RS. Falls in the elderly: assessment of prevalence and risk factors. Pharmacy practice. 2018;16:1206.

32 Aleid A, Shuiel HKB, Alyabis NA, Alfaraj AH, Dahlan DJ, Alkhatib FM, et al. Predictors and outcomes of falls in older adults presenting to the emergency room in Saudi Arabia: a cross-sectional analysis. Cureus. 2023;15.

33 Alqurayshah ANH, Alhendi SSO, Aloqil AMM, Alqurayshah MAA, Alrawas NAS, Almorshed BM, et al. Prevalence and Determinants of Fall Injury among Elderlies in Najran, Saudi Arabia. Advance in Clinical and Experimental Medicine. 2023;10.

34 Alshehri FH. The effect of COVID-19 on the risk of falling in old age adult patients: a cross-sectional study. European Review for Medical & Pharmacological Sciences. 2024;28.

35 Amin A, Mohammed ZA, Amin OSM, Thanoon R, Shareef SH, Oakley TJ, et al. Falls in Older People with Diabetes Mellitus: a study from Kurdistan of Iraq. World Family Medicine. 2019;17:16-21.

36 Stewart BT, Lafta R, Esa Al Shatari SA, Cherewick M, Flaxman A, Hagopian A, et al. Fall injuries in Baghdad from 2003 to 2014: Results of a randomised household cluster survey. Injury. 2016;47:244-9.

37 Muhaidat J, Al-Khlaifat L, Al-Yahya E, Mohammad M, Okasheh R, Qutishat D, et al. Factors Associated with Physical Activity in Jordanian Older People. Activities, Adaptation & Aging. 2022:1-18.

38 Adly NN, Abd-El-Gawad WM, Abou-Hashem RM. Relationship between malnutrition and different fall risk assessment tools in a geriatric in-patient unit. Aging clinical and experimental research. 2020;32:1279-87.
39 al Tehewy MM, Amin GE, Nassar NW. A Study of Rate and Predictors of Fall Among Elderly Patients in a University Hospital. Journal of Patient Safety. 2015;11:210-4.
40 Aly MA, Saber HG. Long COVID and chronic fatigue syndrome: A survey of elderly female survivors in Egypt. International journal of clinical practice. 2021:e14886.
41 Amer MS, Abdellah AF, Elsayed ES. Fear of falls among nursing homes residents and community dwelling elderly. The Egyptian Journal of Hospital Medicine. 2018;73:6785-8.
42 Said MT, Mohsen O, Abozied AM, Salama M. Falls and associated risk factors in a sample of old age population in Egyptian community. Frontiers in public health. 2023;11.
43 El-Gilany A-H, Hatata E-s, Soliman S, Refaat R. Falls among elderly persons living in a rural community setting, Egypt. ME-JAA. 2011
6:18.
44 El-Gilany A-H, Hatata E-s, Soliman SM, Refaat R. Prevention of recurrent falls in elderly: a pre-post intervention study in a rural community, Egypt. International Journal of Collaborative Research on Internal Medicine & Public Health. 2013;5:0-.
45 El-Kawaly WH, Amer MS, Hamza SA, Arif ER. Fracture risk assessment in geriatric homes in Egypt. Indian J Med Res Pharmaceutical Sci. 2016;3:4-14.
46 Khattab Abd El-Rahman S. Impact of Balance Disorders on the Health Outcomes of Elderly People in Damanhur-Egypt. Journal of High Institute of Public Health. 2014;44:41-7.
47 Elsamahy EA, Ebtesam MW, Elsayed S. Risk Factors for fall among Elderly in a Rural Community in El-Monofya Governorate. Saudi Journal of Nursing and Health Care. 2019;2:1-14.
48 Hamed AF, Mohammed NA, Aly H. Elderly Falls Prevalence and Associated Factors in Sohag Governorate. The Egyptian Journal of Community Medicine. 2017;35:1-13.
49 Ismail GM, Fahim HI, Bakr I, Wassif GO, Hamza SA. Risk of falls and Effect of a Health Education Program in Prevention of Falls among Elderly in Geriatric Homes in Cairo, Egypt. The Egyptian Journal of Geriatrics and Gerontology. 2018;5:1-7.
50 Kamel MH, Abdulmajeed AA, Ismail SS. Risk factors of falls among elderly living in urban Suez--Egypt. The Pan African medical journal. 2013;14:26.
51 Khater MS, Mousa SM. Predicting falls among Egyptian nursing home residents: A 1-year longitudinal study. Journal of clinical gerontology and geriatrics. 2012;3:73-6.
52 Mabrouk A, Maher A, Nasser S. An epidemiologic study of elderly burn patients in ain shams university burn unit, cairo, egypt. Burns. 2003;29:687-90.
53 Mahmoued SB, Abd Allah ES, El-Naggar SAE-M. Prevalence of Falling among Elderly at Home in Rural Area of Zagazig District. Zagazig Nursing Journal. 2014;10:18-35.
54 Makhlouf MM, Ayoub AI. Falls among institutionalized elderly in Alexandria. J Egypt Public Health Assoc. 2000;75:507-28.
55 Mohamed AA, El-Shamaa ET, Mohamed JAE-R. Assess the effect of exercises program on balance and prevention of recurrent falling among elderly people. Minia Scientific Nursing Journal. 2019;6:63-70.
56 Mohammed AE, Orabi EE, Hennery H. Falls among the elderly in rural areas of the Sharkia Governorate, Egypt. Turkish Journal of Public Health. 2018;16:214-22.
57 Mohammed RA, Nour-Eldein H, El Din Abdel-Halim AW, Abdulmajeed AA. Effect of a fall prevention program for elderly persons attending a rural family medicine center, Egypt. Journal of Public Health-Heidelberg. 2019;27:301-8.
58 Mohammed RF, Alagamy ZG, El-saidy TM. Detection of the Modifiable and Non-Modifiable Risk Factors and the Adverse Consequences of Falling among Elderly People. Assiut Scientific Nursing Journal. 2021;9:166-78.

59 Mohsen MM, Sabola NE-S, Abd El-Salam EA, Matar HL. Effect of Environmental Home Modification on Falling Risk Reduction among Elderly in a Rural Community. 2019.
60 Sakr K, Farag IA, Zeitoun IM. Review of 509 mandibular fractures treated at the University Hospital, Alexandria, Egypt. The British journal of oral & maxillofacial surgery. 2006;44:107-11.
61 Saleh NMH, Ibrahim HS, Mohamed HNAE-A, El-Gilany A-H. Predictors of fear of falling among community dwelling older adults in Mansoura City, Egypt. International journal of Nursing Didactics. 2018;8:63-71.
62 Saudi RA, Eman E Tosson, and Hend M Salama. Association between frailty status and cognitive performance of elderly patients attending the Family Medicine Outpatient Clinic in Ismailia, Egypt. Family Medicine & Primary Care Review. 2021;23:232-8.
63 Mervat AS, Soha, Kamel. Mesbah, Mohammed, Masoud. Risk Factors Regarding Fall Among Elderly Clients at Geriatric Homes. 2015.
64 Sharaf AY, Ibrahim HS. Physical and psychosocial correlates of fear of falling among older adults in assisted living facilities. Journal of Gerontological nursing. 2008;34:27-35.
65 Tawfik HM, Desouki RR, Singab HA, Hamza SA, El Said S. Multidimentional Preoperative Frailty Assessment and Postoperative Complication Risk in Egyptian Geriatric Patients Undergoing Elective Cardiac Surgery. Journal of Alzheimer's Disease. 2021;82:391-9.
66 Hoda W, Samia A-R, Ahmed M. Handgrip strength and falls in community-dwelling Egyptian seniors. Advances in Aging Research. 2013;2013.
67 Bakr IM, Abd Elaziz KM, Elgaafary MM, Kandil SK, Fahim HI. Epidemiologic pattern of falls among inpatients in Ain Shams University Hospitals in Cairo, Egypt. Journal of preventive medicine and hygiene. 2011;52:32-7.
68 Ismail RA, El Sibai RH, Dakessian AV, Bachir RH, El Sayed MJ. Fall related injuries in elderly patients in a tertiary care centre in Beirut, Lebanon. Journal of emergencies, trauma, and shock. 2020;13:142-5.
69 Musharrafieh U, Rahi AC, Taha A, Shamseddine W, Steitieh S, Jamali F, et al. Profile of injured patients presenting to a tertiary hospital in a developing country. Le Journal medical libanais The Lebanese medical journal. 2011;59:191-6.
70 Zrour C, Haddad R, Zoghbi M, Kharsa Z, Hijazi M, Naja W. Prospective, multi-centric benchmark study assessing delirium: prevalence, incidence and its correlates in hospitalized elderly Lebanese patients. Aging clinical and experimental research. 2020;32:689-97.
71 Mahdi MA, Erraoui M, Ngeuleu A, Tahiri L, Rkain H, Allali F. Falls, fear of falling and risk of falls in patients with rheumatoid arthritis: prevalence and associated factors. Pan African Medical Journal. 2017;28.
72 Bachani AM, Ghaffar A, Hyder AA. Burden of fall injuries in Pakistan--analysis of the National Injury Survey of Pakistan. Eastern Mediterranean health journal = La revue de sante de la Mediterranee orientale = al-Majallah al-sihhiyah li-sharq al-mutawassit. 2011;17:375-81.
73 Bibi R, Yan Z, Anwar N, Zeb A, Mian N. Assessment of Fall-Associated Risk Factors in the Muslim Community-Dwelling Elders of Peshawar, Khyber Pakhtunkhwa, Pakistan. Preprintorg. 2023.
74 Fayyaz J, Wadhwaniya S, Shahzad H, Feroze A, Zia N, Mir M, et al. Pattern of fall injuries in Pakistan: the Pakistan National Emergency Department Surveillance (Pak-NEDS) study. BMC emergency medicine. 2015;15:S3.
75 Gul S. Association between risk of fall and physical activity among the elderly population of police colony, Peshawar. Rehman Journal of Health Sciences. 2021;3:97-103.
76 Hashmi Z, Danish SH, Ahmad F, Hashmi M. Falls in geriatric population-A cross sectional study for assessment of the risk factors. Journal of the Dow University of Health Sciences (JDUHS). 2013;7:94-100.

77 Naseer B, Dastgir H, Sadiq A, Salik S, Abid N, Tayyab M. Factors Associated with Risk of Fall in Elderly Population: Risk of Fall in Elderly Population. The Healer Journal of Physiotherapy and Rehabilitation Sciences. 2022;2:221-31.
78 Sikander N, Ahmad T, Shaikh KA, Abid A, Mazcuri M, Nasreen S. Analysis of Injury Patterns and Outcomes of Blunt Thoracic Trauma in Elderly Patients. Cureus. 2020;12.
79 Soomar SM, Dhalla Z. Injuries and outcomes resulting due to falls in elderly patients presenting to the Emergency Department of a tertiary care hospital–a cohort study. BMC emergency medicine. 2023;23:1-10.
80 Tariq F, Nafees T, Khan A, Mustafa A, Ghani S, Munir S. Falls in elderly people: Frequency, Risk factors and Risk assessment (Far study). Osteoporosis International. 2013;24:S565-S.
81 Halaweh H, Willen C, Grimby-Ekman A, Svantesson U. Physical functioning and fall-related efficacy among community-dwelling elderly people. European Journal of Physiotherapy. 2016;18:11-7.
82 Younis R, Younis M, Hamidi S, Musmar M, Mawson AR. Causes of traumatic brain injury in patients admitted to Rafidia, Al-Ittihad and the specialized Arab hospitals, Palestine, 2006?2007. Brain injury. 2011;25:282-91.
83 Braham MY, Gharbeoui M, Bellali M, Attia H, Harzallah H, Bekir O, et al. Unnatural death in the elderly: a retrospective study of medicolegal autopsies in Northern Tunisia. Egyptian Journal of Forensic Sciences. 2018;9.
84 Abdelkhalik AA. Assessment of elderly awareness regarding Balance Disorders and Falls Prevention. Helwan International Journal for Nursing Research and Practice. 2023;2:145-59.
85 Fahim DFM, Samy HM, Yousef AK. Proposal for a screening protocol for falls among old subjects attending the audio-vestibular clinic. The Egyptian Journal of Otolaryngology. 2023;39:125.
86 Ibrahim MAA, Ahmed AE-sH. Relationship between Visual Functioning, Balance, and Fear of Falling among Community-dwelling seniors with Cataract. Egyptian Journal of Health Care. 2023;14:807-25.
87 Saad M. Risk Factors of Falling among Elderly People in Suez Canal University Hospitals. Trends in Nursing and Health Care Journal. 2024;8:65-85.
88 Khatib SE, Malham CB, Andrieu S, Strumia M, Cestac P, Salameh P. Fall risk factors among poly-medicated older Lebanese patients in primary care settings: a secondary cross-sectional analysis of the "MGPIDP-L project". BMC Geriatr. 2024;24:327.
89 Ahmed Gaber A, Sayed Abd–Ghafar Mohamed S, Awadeen L, Abd Elkader Ahmed M. The Impact of (Domestic) Falling on General Health Status of elderly in Beni Suef Governorate. Egyptian Journal of Health Care. 2024;15:1646-68.
90 Abdalla S. Patterns of vulnerability to non-fatal injuries in Sudan: initial evidence from a national cross-sectional survey. Injury prevention : journal of the International Society for Child and Adolescent Injury Prevention. 2014;20:310-6.
91 Hoy D, Brooks P, Woolf A, Blyth F, March L, Bain C, et al. Assessing risk of bias in prevalence studies: modification of an existing tool and evidence of interrater agreement. J Clin Epidemiol. 2012;65:934-9.
92 Furuya-Kanamori L, Barendregt JJ, Doi SAR. A new improved graphical and quantitative method for detecting bias in meta-analysis. JBI Evidence Implementation. 2018;16:195-203.
